# An fMRI study of initiation and inhibition of manual and spoken responses in people who stutter

**DOI:** 10.1162/IMAG.a.89

**Published:** 2025-07-31

**Authors:** Charlotte E. E. Wiltshire, Jennifer Chesters, Saloni Krishnan, Gabriel J. Cler, Máiréad P. Healy, Kate E. Watkins

**Affiliations:** Department of Psychology, School of Psychology and Sport Science, Bangor University, Wales, United Kingdom; Wellcome Centre for Integrative Neuroimaging, Department of Experimental Psychology, Radcliffe Observatory Quarter, University of Oxford, Oxford, United Kingdom; NHS Greater Glasgow and Clyde Health Board, Glasgow, United Kingdom; Department of Language and Cognition, Division of Psychology and Language Sciences, University College London, London, United Kingdom; Department of Speech and Hearing Sciences, University of Washington, Washington, DC, United States; Department of Psychology, University of Cambridge, Cambridge, United Kingdom

**Keywords:** functional magnetic resonance imaging, stuttering, developmental, basal ganglia, speech, inhibition, psychological, prefrontal cortex

## Abstract

Stuttering is characterised by difficulties initiating speech and frequent interruptions to the flow of speech. Neuroimaging studies of speech production in people who stutter consistently reveal greater activity of the right inferior frontal cortex, an area robustly implicated in stopping manual and spoken responses. This has been linked to an “overactive response suppression mechanism” in people who stutter. Here, we used fMRI to investigate neural differences related to response initiation and inhibition in people who stutter and matched controls (aged 19–45) during performance of the stop-signal task in both the manual and speech domains. We hypothesised there would be increased activity in an inhibitory network centred on the right inferior frontal cortex. Out-of-scanner behavioural testing revealed that people who stutter were slower than controls to respond to ‘go’ stimuli in both the manual and the speech domains, but the groups did not differ in their stop-signal reaction times in either domain. During the fMRI task, both groups activated the expected networks for the manual and speech tasks. Contrary to our hypothesis, we did not observe differences in task-evoked activity between people who stutter and controls during either ‘go’ or ‘stop’ trials. Targeted region-of-interest analyses in the inferior frontal cortex, the supplementary motor area, and the putamen bilaterally confirmed that there were no group differences in activity. These results focus on tasks involving button presses and production of single nonwords, and therefore do not preclude inhibitory involvement related specifically to stuttering events. Our findings indicate that people who stutter do not show behavioural or neural differences in response inhibition, when making simple manual responses and producing fluent speech, contrary to predictions from the global inhibition hypothesis.

## 1 Introduction

Contemporary evidence suggests developmental stuttering is a neurodevelopmental variation which, at the group level, has a characteristic neural profile (Belyk et al., 2017; Brown et al., 2005; Budde et al., 2014; Chang & Guenther, 2020; Neef et al., 2015; Watkins et al., 2008). There are both functional and structural neural differences within the speech motor control network in people who stutter when compared with people who are typically fluent. One part of this network is the right inferior frontal cortex (IFC), which is overactive in people who stutter during speech production, even when perceptibly fluent, and has been described as a ‘neural signature’ of developmental stuttering (Brown et al., 2005). The right IFC is also a key node in the network of brain areas involved in response inhibition in both manual and speech tasks (Aron et al., 2004; Chambers et al., 2007; Xue et al., 2008). Accordingly, the overactivity of this area described in developmental stuttering may reflect, at least in part, differences in the neural control of action inhibition, specifically an imbalance of excitation and inhibition through the inhibitory cortico-basal ganglia-thalamo-cortical loops, including the right IFC (Alm, 2004; Hartwigsen et al., 2019; Metzger et al., 2018; Neef et al., 2015, 2018). Here, we wished to test whether right frontal overactivity is linked to differences in inhibitory control of speech and hand movements using a classic response inhibition task in people who stutter, first behaviourally, and then while recording functional MRI.

Greater activity in the insular, inferior frontal, and opercular cortex of the right hemisphere during speech production is robustly found in imaging studies of people who stutter relative to controls (Brown et al., 2005; Neef et al., 2015). For example, activity in the right posterior IFC (pars opercularis) was higher in people who stutter compared with controls during an imagined speaking task (Neef et al., 2018). In a separate study, brain activity in a more anterior portion of the IFC (pars orbitalis) during overt sentence reading was negatively correlated with stuttering severity, such that greater activity was associated with fewer stuttered syllables which the authors interpreted as reflecting compensation (Kell et al., 2009). This characteristic overactivity in the right IFC observed during overt and covert fluent speech production in people who stutter disappears when fluency increases after therapy (De Nil et al., 2003; Kell et al., 2009), or during temporary fluency enhancing techniques that eliminate stuttering, such as choral reading (Fox et al., 1996). However, the interpretation and the mechanisms underlying this robust finding of overactivity in the right IFC in people who stutter remain unclear. For example, it is unclear whether functional differences reflect general *traits* of developmental stuttering or specifically relate to perceptible or imperceptible moments of stuttering (*state* level). Recent evidence suggests that greater activation is more associated with state-level stuttering: during dysfluent states relative to fluent ones, there was greater activation of inferior frontal and premotor cortex extending into the frontal operculum, bilaterally (Connally et al., 2018). Meta-analytic techniques applied to imaging studies in people who stutter indicate overactivity in the right frontal opercular and insular cortex in both trait and state analyses (Budde et al., 2014) but only in association with traits in another study (Rolandic operculum (Belyk et al., 2017)).

Similarly, it is unclear whether greater activity in the right frontal regions is related to error detection as a result of a stuttered moment or reflects a hyperactive signal to inhibit movements, such that an anticipated stuttering moment is never realised. The DIVA (Directions Into Velocities of Articulators) model supports the idea that greater activity in right frontal regions relates to error detection, considering the right posterior inferior frontal gyrus/ventral pre-motor areas as a feedback control map (Alm, 2004; Hartwigsen et al., 2019; Metzger et al., 2018; Neef et al., 2018), detecting sensory-motor speech errors (Tourville et al., 2008). In contrast, evidence that right IFC activation reflects overactive response inhibition in people who stutter comes from a study that attempted to isolate the time course of right IFC activity during speech, from initiation to inhibition of the utterance (Neef et al., 2016). Compared with other regions activated by speaking, including the left IFC and temporal areas, the right IFC showed delayed peak activity, corresponding to the end of utterances. This temporal delay was observed in both groups; however, the peaks were larger in the right than left hemisphere in people who stutter. In addition, increased right hemisphere structural connectivity between the posterior IFG pars opercularis, pre-SMA, and subthalamic nucleus was reported in participants with more severe stuttering, as measured by the SSI-4 (Neef et al., 2018). Importantly, hyperactivation in right IFC in people who stutter was associated with imagined speech and humming, as well as the cessation of those tasks (Neef et al., 2018).

The aforementioned evidence cannot distinguish between a causal or compensatory role for the increased activity and connectivity in right IFC. Recently, structural connectivity in a large sample of children who stutter was assessed at the level of three sub-parcels within right IFC, which have been linked to action execution (posterior ventral, superior), action inhibition (posterior ventral, inferior), and spatial attention (posterior dorsal) (Hartwigsen et al., 2019). While there were no group-level differences in connectivity profiles between children who stutter and controls, boys who stutter had increased connectivity profiles compared with girls who stutter and control boys in the parcels linked to action execution and inhibition (Neef et al., 2023). Boys are more likely to continue to stutter compared with girls and, therefore, these results may link to the continuation of stuttering into adulthood. Correlations between connectivity and SSI-4 score were not significant, however. Combined, these results indicate that inhibition of actions, including imagined actions in both the speech and manual domains, may be linked with developmental stuttering. These results are in accord with the overactive inhibition hypothesis, which proposes that increased activation in right IFC represents an increased tendency to globally (i.e., broadly, and non-specifically) inhibit motor responses via the cortico-basal ganglia-thalamo-cortical inhibition loops, which contributes to stuttering when speech is the intended motor act (Neef et al., 2016, 2018).

The right IFC is part of a cortico-subcortical network of areas that controls movement initiation and inhibition (Aron et al., 2004; Aron & Poldrack, 2006). The hyperdirect pathway through the basal ganglia involves direct input from the IFG to the ventral subthalamic nucleus (STN) bypassing the striatum (Chen et al., 2020). As the excitatory input from the cortex increases STN firing, which, in turn, excites the inhibitory output of the internal segment of the globus pallidus, the hyperdirect pathway is thought to provide rapid inhibition of basal ganglia output to the motor cortex (Nambu et al., 2002). In vivo studies of the human brain using diffusion imaging identified white matter tracts connecting the STN with the supplementary motor complex (pre-SMA/SMA) and right IFG (Aron et al., 2007), which are connected, in turn, via the frontal aslant tract. The involvement of the right IFG in stopping movement is supported by functional imaging results indicating that activity in the right IFG and the STN region is associated with individual differences in movement inhibition (Aron & Poldrack, 2006); specifically, greater activity was associated with faster stopping times.

Based on this evidence, it is theorised that the output from the thalamus fails to provide appropriate timing cues for the initiation of speech movements to the motor networks, including SMA, premotor/motor cortex, and cerebellum (Alm, 2004; Aron et al., 2004; Mink, 1996). Further evidence in support of a role for the basal ganglia or dopamine (or both) in developmental stuttering comes from pharmaceutical and lesion studies. Dopamine antagonists appear to improve fluency (Maguire et al., 2000), whereas agonists worsen fluency (Anderson et al., 1999). In addition, increased levels of dopaminergic activity were described in the network of areas implicated in stuttering, including the medial prefrontal cortex, orbitofrontal cortex, insular cortex, auditory cortex, as well as the ventral limbic cortical regions (Wu et al., 1997). More recently, people who stutter were found to have elevated iron concentrations, which is linked to increased dopamine, in the basal ganglia (Liman et al., 2021), as well as the left hemisphere speech motor network (Cler et al., 2021). Patients with neurogenic (acquired) stuttering have lesions encompassing the putamen (striatum), pallidum, cortical motor areas (Heuer et al., 1996), and the thalamus (Van Borsel et al., 2003). While such lesion studies suggest a link between the basal ganglia-cortical circuits and stuttering, the size of the lesions makes it difficult to establish a causal relationship between damage to a specific region and behaviour (Alm, 2004). A key piece of evidence that is missing is whether people who stutter show greater activity in this “stopping” network during an inhibition task, independent of speech and stuttering.

Limited evidence exists directly linking stuttering with differences in neural inhibition networks. One study recorded functional MRI while people who stutter performed a manual GO/NOGO task (Metzger et al., 2018). This study found increased activity in people who stutter relative to controls in the basal ganglia and thalamus and particularly in the substantia nigra during response preparation. Task-related activity in the substantia nigra correlated positively with the trait of stuttering severity. In addition, task-related activity in the globus pallidus and the thalamus showed increased network synchronization with activity in the IFG in people who stutter compared with controls (Metzger et al., 2018). Recent work using magnetoencephalography shows evidence that part of the inhibition network (right pre-SMA) has increased power in the beta domain prior to the onset of an anticipated stuttered word, demonstrating a link between the cognitive aspects of stuttering (anticipation) and reactive inhibitory control (Orpella et al., 2024).

The stop-signal task paradigm has been used in conjunction with fMRI to robustly isolate networks that are involved in response inhibition (Aron & Poldrack, 2006; Chevrier et al., 2007; Ray Li et al., 2008; Xue et al., 2008). During this task, participants produce a button-press response to a visual stimulus as quickly as possible. On a small percentage of randomly inserted trials, participants are cued to inhibit their response by an auditory tone (the “stop signal”). The timing of the stop signal is adjusted for each individual to determine the interval needed such that participants fail to inhibit their action 50% of the time and allow estimation of a stop-signal reaction time (SSRT; the minimum time needed to successfully inhibit a response). This paradigm was adapted to measure inhibition of speech responses and used to demonstrate that both manual and spoken response inhibition evoke common neural activity in the right IFG (Xue et al., 2008). This suggests that inhibitory control is a domain general process.

The behavioural version of the stop-signal task has been used to test manual response inhibition in adults and children who stutter. Adults who stutter showed longer SSRTs (longer stopping times) compared with a control group (Markett et al., 2016; Treleaven & Coalson, 2020), but this result was not replicated in a study comparing the speech and manual domains in people who stutter (Treleaven & Coalson, 2021). In the latter study, there were no group differences for inhibition and no relationship between inhibition and stuttering severity (SSI-4). There was, however, a positive relationship between speech inhibition measured using a speech stop-signal task and scores on the Overall Assessment of the Speaker’s Experience of Stuttering (OASES), suggesting that speech inhibition may be linked to an adverse experience of stuttering. Another study using the SSRT task showed no differences in inhibition between children who do and do not stutter (Eggers et al., 2018). These inconsistent findings do not provide a clear developmental trajectory of inhibitory control in children and adults who stutter. Behaviourally, the evidence to support the global inhibition hypothesis, namely that people who stutter show an overly involved inhibitory response via the cortico-basal ganglia-thalamo-cortical pathways (Neef et al., 2018) regardless of domain, remains equivocal. It is clear, therefore, that the addition of fMRI recording during the stop-signal task in both manual and speech domains could help to further understand the neural basis of response inhibition in people who stutter.

Here, we used fMRI during a stop-signal task to capture both manual and speech initiation and inhibition responses of people who stutter and controls. Our aim was to investigate neural differences related to response initiation and inhibition in people who stutter using the stop-signal paradigm. Based upon evidence linking greater right IFC activity with shorter stopping responses (SSRT; e.g. Aron & Poldrack, 2006), in combination with the observation that people who stutter have greater activity in right IFC (e.g. Belyk et al., 2017), the global inhibition hypothesis would predict shorter SSRT behaviourally and greater activation of the right hemisphere inhibition network during ‘go’ and ‘stop’ trials in people who stutter. In contrast with this hypothetical pattern of results, recent behavioural work reported longer stopping reaction times in people who stutter (Markett et al., 2016; Treleaven & Coalson, 2020). An imbalance in the inhibition network (the basal ganglia-thalamo-cortical loops) could lead to either longer or shorter SSRTs. Here, we combined behavioural responses with fMRI to investigate the role of the inhibition network in people who stutter. Using both the manual and speech versions of the stop-signal task allowed us to address whether differences in inhibitory control in people who stutter might be considered domain general or domain specific.

### 1.1 Procedure

All participants completed manual and speech versions of the stop-signal task outside the scanner in a pre-scan behavioural session. This allowed us to titrate the timing of the stop-signal for each participant to be used in the in-scanner tasks performed while we acquired fMRI data. We describe the behavioural session followed by the fMRI session.

## 2 Pre-Scan Behavioural Task

### 2.1 Participants

Forty-seven adults who stutter and 23 controls participated in the behavioural study. Participants were recruited from the community through advertisement, social media, or word of mouth. Groups were balanced for gender, age, years of education, and ethnicity (see Table 1, below). Participants spoke English as their first language and had no diagnosis of a speech or language disorder other that stuttering. All had normal hearing and normal or corrected-to-normal vision. All people who stutter reported the onset of stuttering during childhood (i.e., before 10 years old) and had not undergone speech therapy for at least 6 months prior to testing.

**Table 1.** Demographics of participants for the pre-scan behavioural SSRT task.

	Control		Stutter	
Condition (N participants included in analysis)	Manual (N = 23)	Speech (N = 20)	Manual (N = 46)	Speech (N = 34)
N participants excluded^1^	0	3	1	13
Gender				
Man	18 (78 %)	16 (80 %)	39 (85 %)	30 (88 %)
Woman	5 (22 %)	4 (20 %)	7 (15 %)	4 (12 %)
Age (years)				
Median [min,max]	28 [19,44]	29 [19,44]	32 [19,45]	31 [19,45]
Handedness				
Left	1 (4 %)	1 (5 %)	3 (7 %)	1 (3 %)
Right	22 (96 %)	19 (95 %)	43 (93 %)	33 (97 %)
SSI score				
Median [min,max]	NA	NA	28 [16,40]	28 [16,40]

1 Participants excluded from the original sample size of 23 controls and 47 people who stutter. See text for a detailed description.

In the stuttering group, participants were included if their stuttering severity ranged from “moderate” to “very severe” as measured by the SSI (Stuttering Severity Instrument-4; Riley 2009). Recruitment was part of another study that involved an intervention to enhance fluency; therefore, we did not recruit those in the mild range of stuttering severity. Two individuals (one a qualified speech-language pathologist/therapist) independently scored stuttering severity for a subset of 24 participants who stuttered. Inter-rater reliability was using the Intraclass Correlation Coefficient (ICC) with a two-way random-effects model (‘ICC’ function from ‘Psych’ package, R), and showed excellent inter-rater reliability (single ratings: 0.97; average ratings 0.99). Participants spoke English as their first language.

One participant (person who stutters) did not complete the manual version of the behavioural task. During the speech version of the pre-scan behavioural task, a technical error involving the microphone occurred for 16 participants (13 people who stutter, 3 controls) which led to the exclusion of behavioural data from these participants. Therefore, behavioural data from 46 people who stutter and 23 controls were included in the analysis of the manual task and from 34 people who stutter and 20 controls in the analysis of the speech task (see Table 1).

The University of Oxford Central University Research Ethics Committee approved the study. Written informed consent was obtained from all participants for being included in the study, in accordance with the Declaration of Helsinki, and with the procedure approved by the committee.

### 2.2 Pre-scan task

The stop-signal task was run before the scan session in order to determine the stop-signal delay (SSD) to be used during scanning for each participant. The tasks were delivered using MATLAB (MathWorks Inc, R2016a) and were identical to the pre-scan tasks described in Xue et al. (2008). 240 Go trials and 80 Stop trials were used to estimate the SSD for manual responses. On Go trials, each trial started with a white fixation cross, presented on a grey background for 500 ms, after which a visual stimulus appeared on the screen for 1 s. For the manual task, participants were presented with a left or right facing arrow (< or >). Participants responded by pressing one of two buttons with the right index finger corresponding to the left/right direction of the arrow. For the speech task, participants were presented via headphones with a one-syllable pseudoword which followed English phonology (e.g., “bafe”, “seeth”). Pseudowords were unique to each trial (i.e., not repeated). Participants responded by saying the pseudoword out loud. Speech was recorded via a microphone (Samsung C01U Pro condenser microphone) and was automatically detected (maximum signal amplitude during trial greater than 0.01 normalised floating-point values [-1,1]). An investigator was also in the room to monitor responses for stuttering during the task. Participants were not observed to stutter (produce audible prolongations or repetitions of sounds or syllables, or inaudible blocks). The very high responses rate on “Go” trials for both groups (see results) indicates that the nonword task did not lead to stuttering in people who stutter. The inter-trial delay was 2 s. Stop trials were visually identical to go trials except that an auditory cue (500 ms) was played via over-ear headphones at an interval after presentation of the visual stimulus (arrow/pseudoword). Participants were instructed to respond as fast as possible to the visual stimulus and were told that it would not always be possible to stop in response to the auditory cue. The delay was changed adaptively according to the participant’s behaviour. If the participant inhibited successfully on a stop trial, then successful inhibition was made less likely on the next stop trial by increasing the SSD by 50 ms, thus increasing the time between the onset of the visual stimulus and the presentation of the stop signal and the likelihood that the participant would begin to respond before the auditory stop signal was presented (i.e., making the stopping more difficult). If the participant failed to inhibit, the SSD was decreased by 50 ms, thus giving participants less time to initiate a Go response before the cue was played and the response was more easily stopped or inhibited. The SSD was varied in this way using a staircase algorithm. An individual participant’s SSD was computed as the SSD at which the probability of a participant successfully inhibiting on a Stop trial was 50%. This SSD was used during scanning, thereby ensuring that the task was equally difficult for each participant. As SSD was varied to yield a 50% chance of stopping, the stop-signal reaction time (SSRT) was estimable by subtracting the mean SSD from the median reaction time (RT) of correct Go responses (Go Reaction Time). The SSRT provides a measure of the speed of the stopping process: a short SSRT indicates a quick stopping process, and a long SSRT indicates a slow stopping process.

### 2.3 Behavioural results

**Fig. 1.**
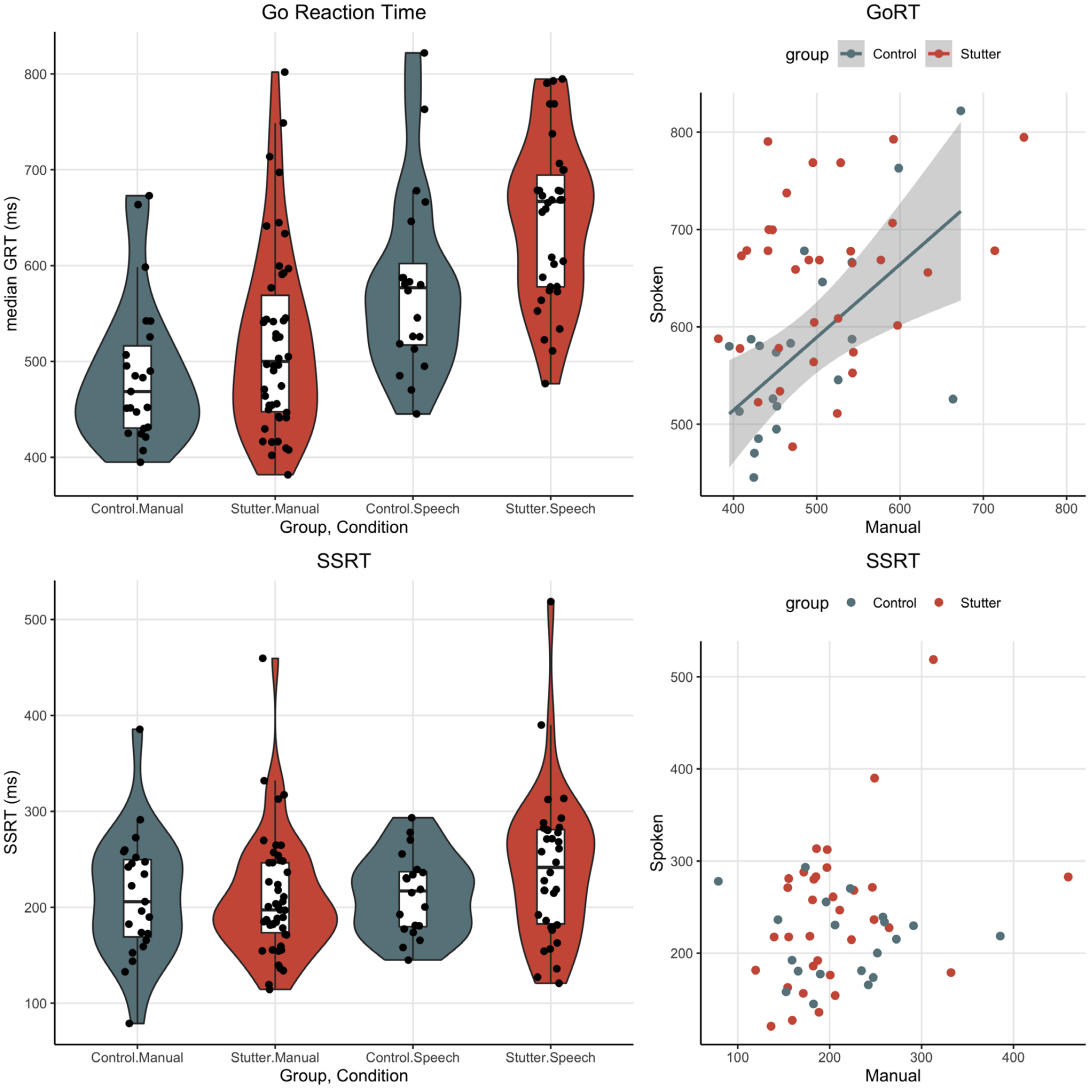
Group performance on the behavioural stop-signal tasks. Left: Violin and box plots are shown for the people who stutter (orange) and control (grey) groups by task for Go Reaction Time (top panel) and Stop Signal Reaction Time (bottom panel). Data points for individual subjects are shown by the black dots. The group median is indicated by the horizontal solid black line and the interquartile range by the white shaded box. The colour shading indicates the distribution of data for each group. Right: The relationship between the two tasks for Go Reaction Time (top panel) and SSRT (bottom panel). The figure shows the relationship between Manual and Spoken values for people who stutter and controls. Each data point corresponds to a participant’s measurements in both manual and spoken tasks. Where significant, the plot highlights the linear trend through the data points using a regression line (solid line), indicating the general direction of the relationship between the two tasks.

The results of the pre-scan behavioural study are summarised in Table 2 and visualised in Figure 1.

**Table 2.** Group performance on the stop-signal behavioural task.

	Control		Stutter	
	Manual (N = 23)	Speech (N = 20)	Manual (N = 46)	Speech (N = 34)
% Go response				
Median [min,max]	98.75 [97.08,100.0]	95.42 [66.67,100.0]	98.33 [76.67,100.0]	94.79 [64.17,100.0]
Go reaction time (ms)				
Median [min,max]	468.6 [394.9,672.9]	576.9 [445.2,821.9]	500.0 [381.8,801.9]	667.0 [476.9,794.7]
Stop signal delay (ms)				
Median [min,max]	282.5 [121.3,490.0]	350.0 [215.0,582.5]	305.0 [81.25,687.5]	396.3 [230.0,580.0]
SSRT (ms)				
Median [min,max]	205.8 [78.87,385.6]	216.9 [145.0,293.3]	197.2 [114.4,459.5]	241.6 [120.8,518.7]

The *brms* package (Bürkner, 2017) was used to fit a Bayesian regression model for Go Reaction Time and Stop Signal Reaction Time. For each model, we tested whether the variable could be predicted by task (manual, speech), group (stutter, control) or the interaction between them. Each model included random intercepts for participants, which captures the variation in the measured variable that is unique to each participant. We used mildly informative priors which were set based on expectations from previous literature (Xue et al., 2008). A shifted lognormal posterior distribution gave the best fit to the models, which is typical for reaction time data. These models fitted well with *rhats* uniformly at 1, stationary and well mixing chains with a large number of samples (see R code for details of diagnostics). For each model, we report the Beta coefficient (β), which is the strength of the relationship between the variables of interest, and the credible interval of the beta coefficient, which is the plausibility of finding a range of beta values. Credible intervals that do not cross zero indicate a strong effect.

In the first model, the median reaction time on “Go” trials was predicted by task (β = 0.39; lower 95% credible interval (CI) = 0.19, upper 95% CI = 0.65), with the speech condition having longer reaction times compared with the manual condition. There was a small effect of group (β = 0.13; CI = -0.04–0.32) and the interaction between group and task (β = 0.10; CI = -0.11–0.32), with the stuttering group having longer reaction times than the control group, particularly on the speech task. The posterior distribution crosses zero for these latter two effects; however, the probable mass of the posterior distribution was weighted above zero with an estimated probability of an effect above zero of 93% for the effect of group and 83% for the interaction between group and task. This means that, given this model and data, there is a 93% and 83% probability, respectively, that the true effect is not null. In summary, both groups had longer reaction times for spoken compared with manual responses, and this effect was more pronounced in the stuttering group, who, across tasks, had longer reactions times than controls.

In the second model, the Stop Signal Reaction Time was not predicted by any of the factors (task: (β = -0.01; CI = -0.21–0.20; group: β = 0; CI = -0.18–0.18; interaction between group and task: (β = 0.08; CI = -0.17–0.33)). The β values are near zero and the distribution of the posterior values is spread around zero, indicating that given this model and data, the true effect is null. In summary, there were no group or task differences on the stop signal reaction time measure.

Due to the presence of potential outliers, non-parametric Spearman’s rank correlations were used to assess the relationship between the manual and speech tasks for the Go Reaction Time and SSRT, in turn (see Fig. 1, right column). There was a relationship between the Go Reaction Time in the speech and manual conditions for the control group (rho = 0.58, *p* = .008). This suggests that if a control participant was slower to respond on the manual task, they were also slower to respond on the speech task. This relationship was not significant for the group who stutter. For inhibition responses (SSRT), the relationship between speech and manual conditions was not significant for either group. For the stuttering participants only, Spearman’s rank correlations were used to assess the relationship between SSI score and the measured variables (Go Reaction Time and Stop Signal Reaction Time) and resulted in no significant correlations for speech and manual tasks.

## 3 Task fMRI

### 3.1 Participants

Of the initial 47 people who stutter and 23 control participants who took part in the behavioural sessions, 6 participants (5 who stutter and 1 control) did not take part in the manual version of the MRI session, and 7 participants (6 who stutter and 1 control) did not take part in the speech version.

The SSD from the behavioural task was used during scanning to approximate the delay at which participants would successfully stop 50% of the time and fail to stop 50% during stop trials. This approximation did not always result in a near-equal distribution of stopping responses during scanning. Therefore, data were excluded in the following MRI analysis for participants who did not produce at least 10/24 successful or unsuccessful stops during the scan.

For the manual task, two participants (from the stuttering group) were excluded based on this criterion. In addition, data from six participants (people who stutter) were excluded for technical reasons (e.g., button box failure, scanner issues) and a further three participants (one person who stutters and two controls) were excluded for excessive movement during scanning (>2 mm average absolute movement).

For the speech task, 14 participants were excluded because they did not have at least 10/24 successful or unsuccessful stop trials (11 people who stutter and 3 controls). In addition, data from four participants (three from the stuttering group and one control) were excluded for technical reasons (e.g., microphone failure, scanner issues) and a further one control participant was excluded for excessive movement (>2 mm average absolute movement).

The remaining participants are summarised in Table 3 below.

**Table 3.** Demographics of participants for the fMRI SSRT task.

	Control		Stutter	
Condition (N participants included in analysis)	Manual (N = 20)	Speech (N = 17)	Manual (N = 33)	Speech (N = 27)
N participants excluded^1^	3	6	14	20
Gender				
Man	15 (75 %)	12 (71 %)	27 (82 %)	21 (78 %)
Woman	5 (25 %)	5 (29 %)	6 (18 %)	6 (22 %)
Age				
Median [min,max]	27.50 [19.00,44.00]	27.00 [19.00,44.00]	32.00 [19.00,45.00]	31.00 [19.00,45.00]
Handedness				
Left	1 (5 %)	0 (0 %)	2 (6 %)	1 (4 %)
Right	19 (95 %)	17 (100 %)	31 (94 %)	26 (96 %)
SSI				
Median [min,max]	NA	NA	28.00 [16.00,40.00]	28.00 [16.00,40.00]

1 Participants excluded from the original sample size of 23 controls and 47 people who stutter.

N includes participants who did not take part in the MRI session and participants whose data were excluded. See text for a detailed description.

### 3.2 fMRI SSRT task

The stop-signal paradigm was used to assess manual and spoken responses using fMRI as described in Xue et al., (2008). The task comprised 144 Go and 48 Stop trials. The paradigm in the scanner was the same as described above, except that the staircasing algorithm was replaced with a fixed, individualised, SSD, which estimated the probability of successfully inhibiting on 50% of trials.

### 3.3 MRI data acquisition

Image data were acquired using a 3T Siemens magnetom Prisma scanner using a 64-channel head and neck coil. Echo-planar images were acquired with 72, 2-mm isometric slices with a multiband acceleration factor of 8 (TR/TE = 720/33, Flip Angle = 53 deg, FOV = 208, in-plane resolution of 2 x 2 mm). High-resolution T1-weighted structural images of the whole brain were also acquired with an MPRAGE protocol (PAT2, 1 mm isotropic, TR/TE = 2400/3.98). Audio stimuli (i.e., the stop signal) were delivered via OptoActive noise-cancelling headphones, and the participants’ speech was recorded using a noise-cancelling microphone (Optoacoustics ltd, Israel).

### 3.4 Imaging preprocessing and statistics

Data for each participant were preprocessed using standard parameters in FMRIB Software Library (FSL version 6.0.1 http://www.fmrib.ox.ac.uk/fsl [Jenkinson et al., 2012]). For each participant, the whole-brain T1-weighted image was skull stripped using the Brain Extraction Tool (BET; part of FSL). Functional data were processed at the subject-level using FMRI expert analyses tool (FEAT, v 6.0). A temporal high-pass filter with a cut-off of 90s was used to remove low-frequency fluctuations in the signal. Standard motion correction was applied (MCFLIRT). Data were smoothed with a 5-mm full-width-at-half-maximum Gaussian smoothing kernel. B0 unwarping was conducted using the fieldmap images and PRELUDE and FUGUE software running in FSL (Jenkinson et al., 2012). All fMRI volumes were first registered to a reference image (increased SNR and contrast but with same distortions) and then aligned to the individual’s structural scan using brain boundary registration (BBR), implemented using FMRIB’s Linear Image Registration Tool (FLIRT). They were then registered to 2-mm MNI standard space using FMRIB’s Nonlinear Image Registration Tool (FNIRT) for group analyses.

Group comparisons were implemented using FMRIB’s Local Analysis of Mixed Effects stage 1 (Woolrich et al., 2004). For group averages, results are reported using a cluster-forming threshold Z > 3.1, and extent-threshold of *p* < .05 corrected.

We followed up the whole-brain analyses with more constrained ROI analyses. We took an exploratory approach with ROIs from the inhibitory network detailed in Xue et al, including IFG bilaterally, SMA and putamen bilaterally (see Fig. S1). The right lateral occipital cortex was included as a control condition as this area shows a response to the visual stimuli but is not expected to differ between the groups (stutter, control) or the conditions (manual, speech). The percent BOLD signal change for each of the trial types (‘Go’, ‘Successful Stop’, ‘Unsuccessful Stop’) was extracted using Featquery. Masks were created based on the Harvard-Oxford cortical and subcortical probabilistic structural atlases available in FSL thresholded at 50% (i.e., the mask included voxels where the structure was evident in at least 50% of atlas participants). Data from each ROI and trial type were plotted and followed up with Bayesian Regression models using the same approach as the behavioural data, above. Multiple comparisons correction was not applied given the exploratory approach using Bayesian modelling.

### 3.5 fMRI results

The patterns of activation are described for each of the trial types (‘Go’, ‘Successful stop’ and ‘Unsuccessful stop’) followed by a description of the contrast to isolate a stopping response (Successful stop > Go), for each of the conditions in turn.

### 3.6 Go trials

#### 3.6.1 Manual

**Fig. 2.**
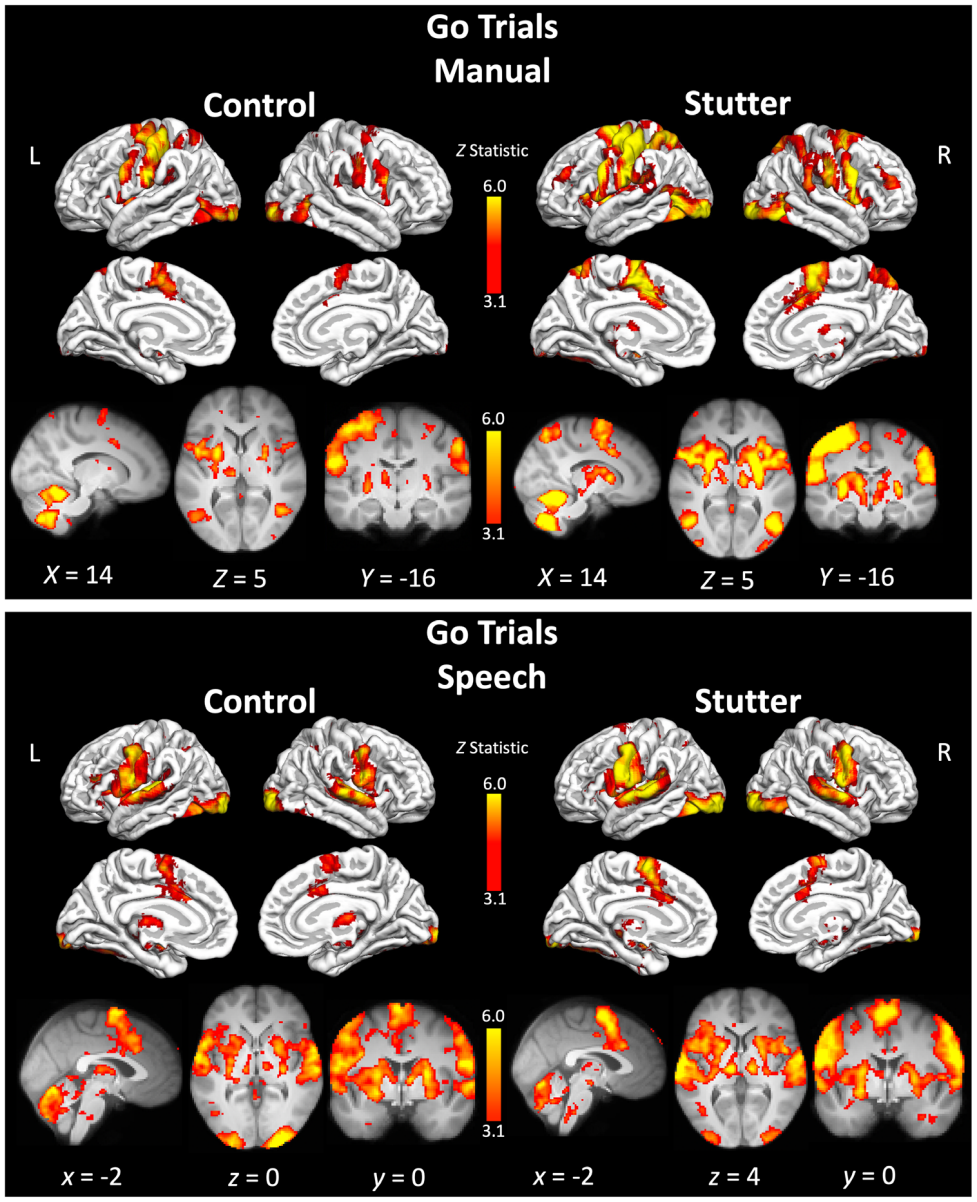
Activity during ‘go’ trials for controls and people who stutter. Top panel shows the manual condition, and bottom panel shows the speech condition. Coloured areas indicate statistical maps (thresholded at Z > 3.1) overlaid on the cortical surface using FreeSurfer or on slices through the brain volume at the coordinate indicated below each image. L – left; R – right. See Supplementary Tables S1 to S4 for a list of areas significantly activated for each group.

Both people who stutter and controls showed activation of the expected cortical and subcortical motor areas, particularly in the left hemisphere, however there were no significant differences in activity between the groups. There was extensive activation of the left precentral gyrus at the level of the hand representation extending to the frontal operculum, insular cortex and inferior frontal gyrus. The right precentral gyrus, extending to the medial frontal and inferior frontal gyrus, was also activated, though to a lesser extent. In addition, there was activation in the SMA extending to the cingulate gyrus, the putamen extending to the opercular cortex bilaterally, and extensively in the cerebellum bilaterally. There was also activity bilaterally in the occipital cortex (see Fig. 2 and Tables S1 and S2). This pattern of activation was consistent with the task which involved a simple button press with the right index finger in response to the visually presented arrow. Even though there were no significant differences between groups, people who stutter showed activity in the thalamus bilaterally, whereas controls showed activity in the right thalamus only. Generally, the group of people who stutter had more spatially extensive task-evoked activity compared with the control group, which may be due to differences between the groups in sample sizes (note that we obtained more data in people who stutter compared with controls). Larger samples typically reduce variance, however, so we interpret the greater spatial extent of the stuttering group’s average activity as indicative of greater within-group variance compared with controls, despite the larger sample size (see Table 3).

#### 3.6.2 Speech

The two groups showed very similar patterns of activity, with no significant differences in activity for the contrast between them. This included bilateral activity in the precentral gyrus, at the level of the face, postcentral gyrus, putamen, thalamus, and cerebellum. Participants also had activity in the left but not the right inferior frontal gyrus. Both groups had activity in the insular cortex bilaterally as well as the SMA extending to the cingulate cortex bilaterally. The lateral occipital cortex was activated bilaterally, presumed to reflect processing of the visual stimulus (see Fig. 2 and Tables S3 and S4).

### 3.7 Successful stop trials

#### 3.7.1 Manual

**Fig. 3.**
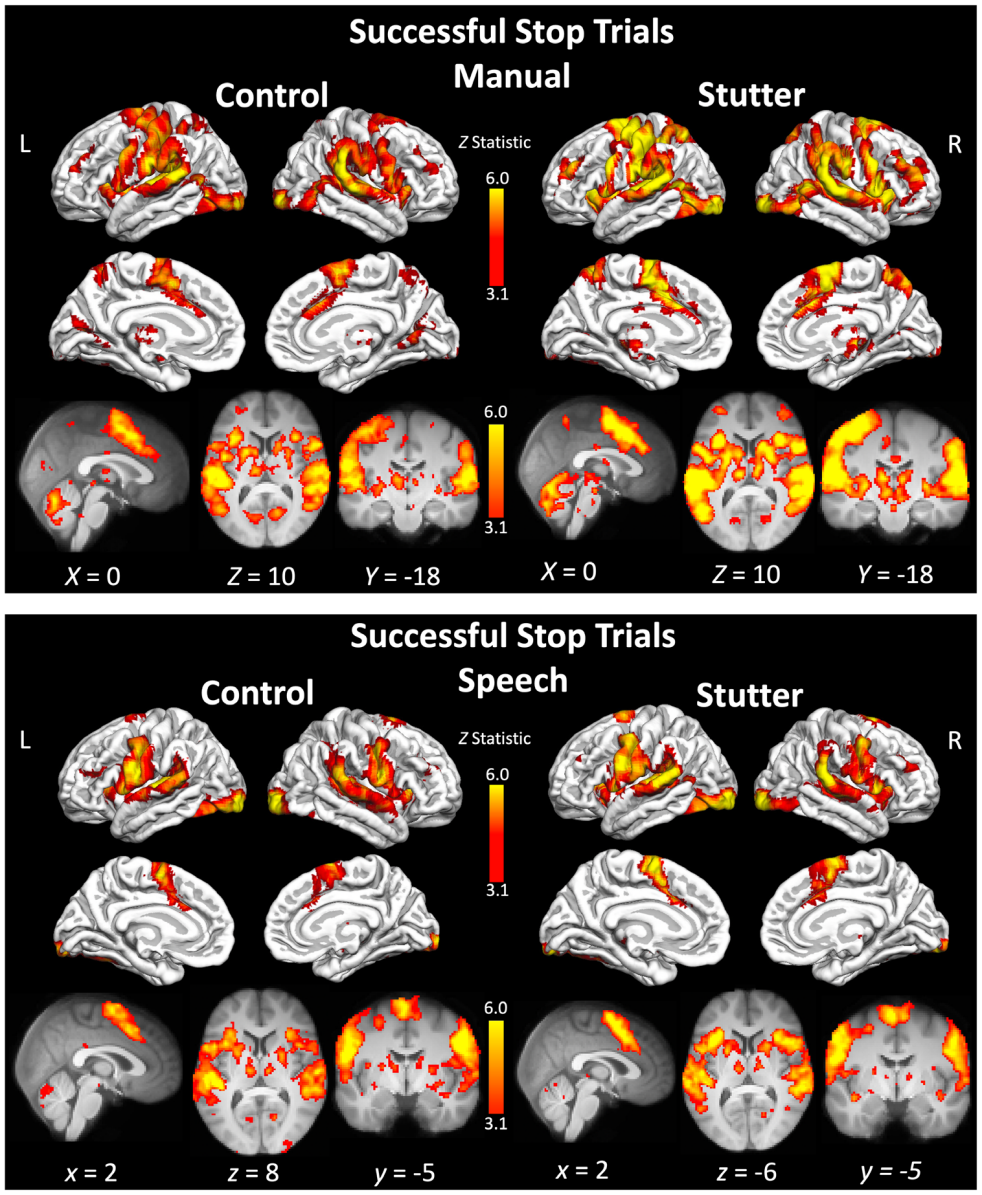
Activity during ‘successful stop’ trials. Coloured areas indicate statistical maps (thresholded at Z > 3.1) overlaid on the cortical surface using FreeSurfer or on slices through the brain volume at the coordinate indicated below each image. L – left; R – right. See Supplementary Tables S5 to S8 for a list of areas significantly activated for each group.

There were no significant differences in activity between the groups. The two groups showed similar patterns of activity for the trials in which the participants heard the stop signal and were successful in stopping themselves from pressing a button with their right finger (see Fig. 3 and Supplementary Tables S5 and S6). Activity on successful stop trials included the pre-and post-central gyri extending to the frontal operculum, insular cortex, and inferior frontal gyrus bilaterally, although the activation was more widespread in the left hemisphere and also included the hand representation of the precentral cortex. This is consistent with participants stopping themselves from moving their right index finger at the “stop” signal. Both groups had extensive activation in the supplementary motor area and cingulate gyrus. Both groups activated the putamen and thalamus bilaterally. Two clear clusters of activity were seen in the planum temporale extending to Heschl’s gyrus, which is consistent with participants hearing an auditory “stop” cue.

#### 3.7.2 Speech

The two groups showed very similar patterns of activity, with no significant differences in activity for the contrast between them. The network of activity for the successful stop trials in the speech condition was similar to that of the hand condition, above (see Fig. 3 and Supplementary Tables S7 and S8). A key expected difference was that the activity in the precentral gyrus bilaterally was located more ventrally at the level of the sensorimotor representation of the face, rather than the hand. This is consistent with participants preparing to execute a vocal response (speak a nonword) and then successfully stopping their response after hearing an auditory cue.

### 3.8 Unsuccessful stop trials

#### 3.8.1 Manual

**Fig. 4.**
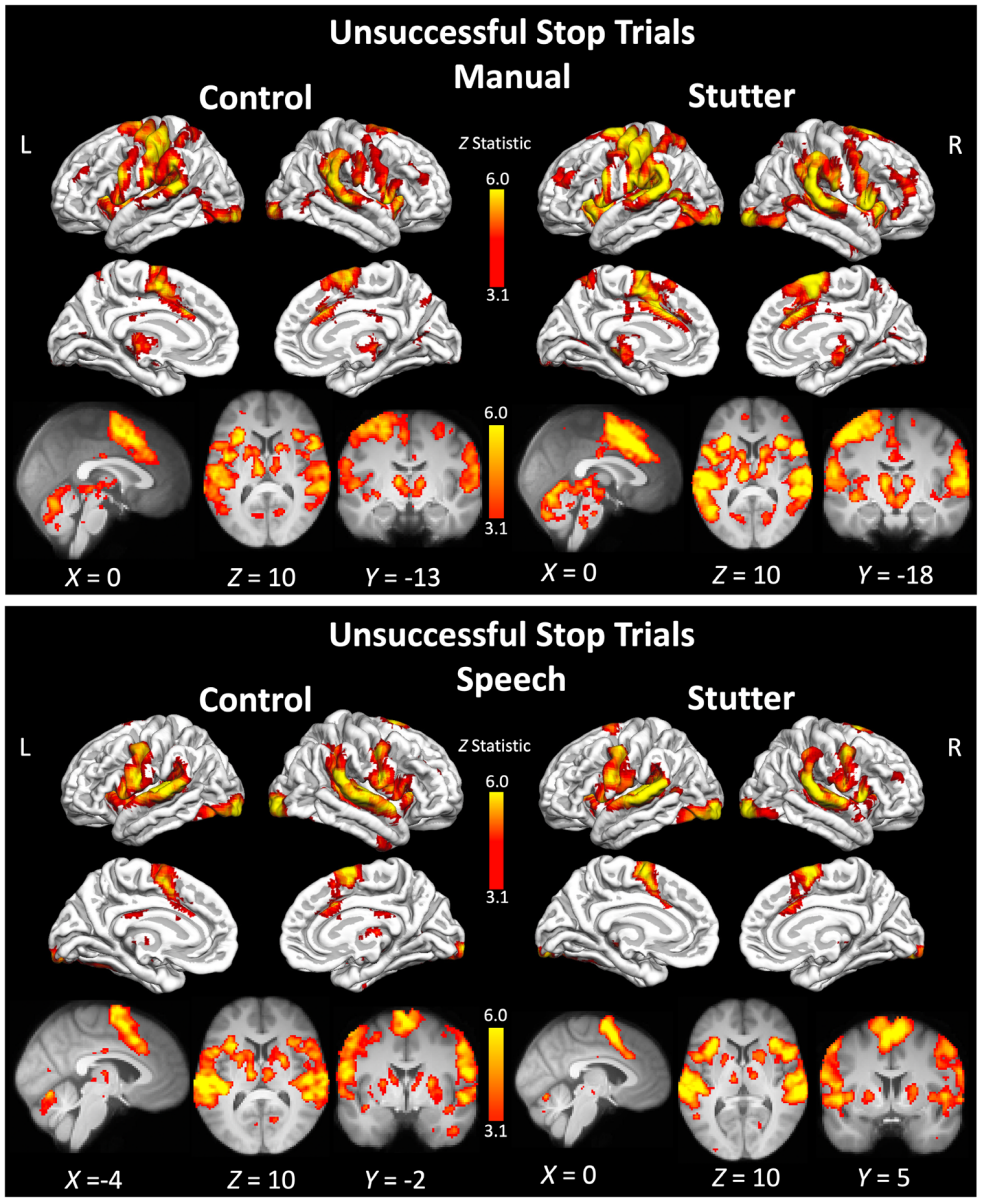
Activity during ‘unsuccessful stop’ trials. See legend to Figure 2 for details. See Tables S9 to S12 for a list of areas significantly activated for each group.

There were no significant differences between the groups. For the ‘unsuccessful stop’ trials, both groups showed widespread activity in the pre- and postcentral gyri bilaterally, with left-lateralised activation at the level of the hand representation. There was extensive activation in the SMA extending to anterior paracingulate and cingulate cortex as well as the cerebellum, bilaterally. There was also extensive activation from the supramarginal gyrus, planum temporale, Heschl’s gyrus, and the superior temporal gyrus bilaterally, consistent with a response to the auditory “stop” cue. Both groups showed bilateral activation of the thalamus, putamen, and caudate nucleus. See Figure 4 and Supplementary Tables S9 and S10.

#### 3.8.2 Speech

For ‘unsuccessful stop’ trials, both the people who stutter and the control group activated a similar network of areas to each other (see Fig. 4 and Supplementary Tables S11 and S12) and there were no significant differences between them. This network included the precentral gyrus at the level of the face extending to the inferior frontal gyrus, frontal operculum, and insular cortex bilaterally. The medial frontal cortex, including the SMA, pre-SMA, and cingulate cortex, also showed activity. There was also extensive activation from the supramarginal gyrus, planum temporale, Heschl’s gyrus, and the superior temporal gyrus bilaterally. The control group showed slightly more widespread activation in the putamen, thalamus, and cerebellum, all bilaterally, in comparison with the group of people who stutter.

### 3.9 Successful stop > Go trials

#### 3.9.1 Manual

**Fig. 5.**
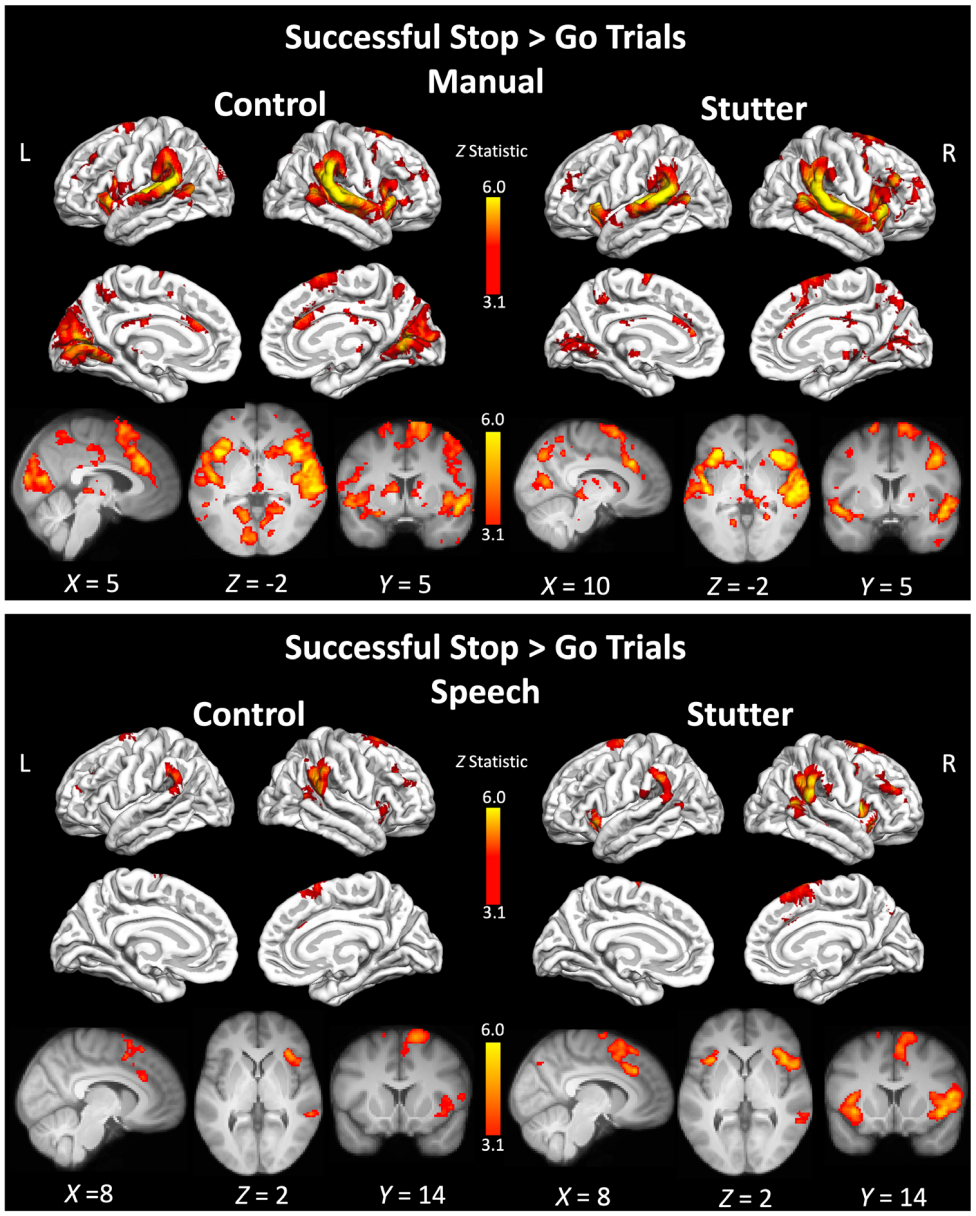
Activity for the contrast Successful stop > Go. See legend to Figure 2 for details. See Supplementary Tables S13 to S16 for a list of areas significantly activated for each group.

There were no significant differences in activation between people who stutter and the control for this contrast between successful stop trials and go trials. Both groups activated the inferior frontal cortex, caudate nucleus, supramarginal gyrus extending to the superior temporal areas, planum temporale, and Heschl’s gyrus, all bilaterally. The pre-supplementary motor area was also activated (see Fig. 5 and Supplementary Tables S13 and S14).

#### 3.9.2 Speech

There were no significant differences between the groups for the speech condition. Both groups activated a more focal network compared with the manual condition. This included the superior temporal gyrus extending to the planum temporale and Heschl’s gyrus, likely in response to the auditory stop cue. In addition, both groups activated the pre-supplementary motor area extending to the cingulate and paracingulate gyri. People who stutter activated the insular cortex extending to the opercular cortex and inferior frontal gyrus (pars opercularis) bilaterally, whereas the control group activated these regions in the right hemisphere only. See Figure 5 and Supplementary Tables S15 and S16 for details.

### 3.10 Exploratory region of interest analysis

The whole-brain analyses detailed above revealed no significant differences between the group of people who stutter and the control group for any of the trial types (‘Go’, ‘Successful Stop’, ‘Unsuccessful Stop’) or the contrasts between them (Successful stop > Go, Unsuccessful Stop > Successful Stop, Successful Stop > Unsuccessful stop). Despite this, some key visual differences remained, particularly pertaining to the inhibitory and speech networks. To ensure we did not make a false negative error due to the stringent statistical thresholding implemented in our whole-brain analyses, we constrained our analyses to five regions of interests (ROIs) from the inhibitory control network, comprising IFG (pars opercularis) bilaterally, putamen bilaterally, and the SMA. We also used the right lateral occipital cortex as a control region as this area was expected to activate in response to the visual stimuli but was not expected to show differences between groups or condition. The ROI masks are shown in Supplementary Figure S1.

For each trial type (‘Go’, ‘Successful Stop’, ‘Unsuccessful Stop’), and the contrast ‘Successful stop > Go’, a Bayesian regression model was run for each of the ROIs in turn with factors of group, condition, and the interaction between them. Participant was added as a random factor. Note that because several participants did not complete both conditions (see Table 3), including participant as a random factor, means that the missing observations are estimated alongside the model parameters during the Markov chain Monte Carlo (MCMC) sampling process. This approach allows for the estimation of posterior distributions that incorporate uncertainty about the missing values, which were considered missing at random.

**Fig. 6.**
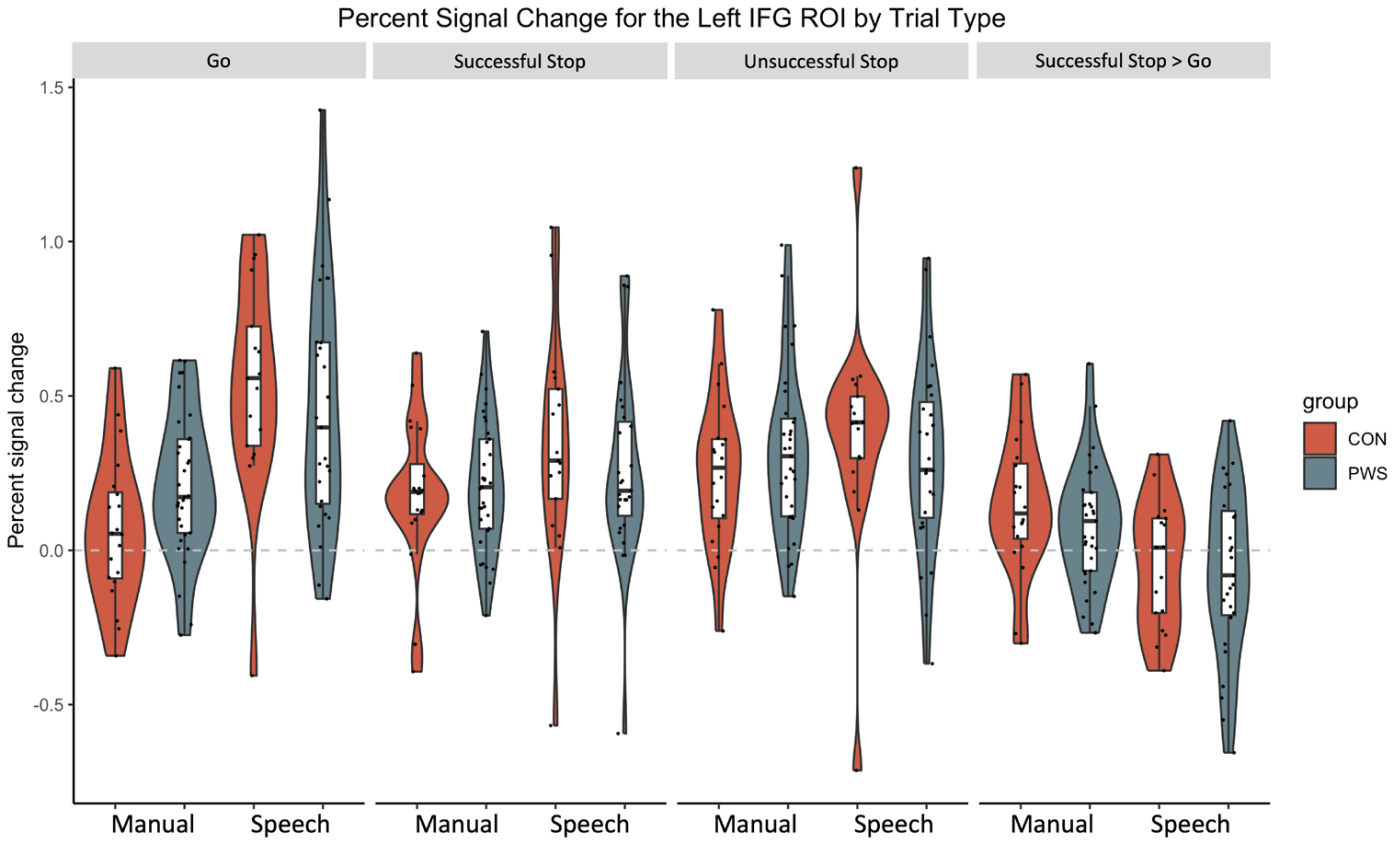
Percent signal change for the left IFG region of interest. The group median is indicated by the solid black line quartiles by the white shaded box; whiskers show values within 1.5 times the IQR. Horizontal grey dotted line represents no signal change (0%).

Percent signal change data from the left inferior frontal gyrus ROI showed consistent effects (Fig. 6) but none were observed for the other ROIs, including the right IFG. For the left IFG and the Go trials, there was an effect of condition, with greater activity for the speech condition compared with the manual condition (β = 0.47; CI = 0.28–0.67), as well as small effects of group (people who stutter activated more than controls; β = 0.14; CI = -0.03–0.32) and the interaction between condition and group (controls showed a larger increase in activation between the hand and speech conditions compared with the people who stutter; β = -0.22; CI = -0.47–0.02). Small effects of condition (more activation for the speech condition compared to the manual condition) were also seen for the successful stop (β = 0.15; CI = 0–0.30) and unsuccessful stop trials (β = 0.12; CI = -0.01–0.26) in the left inferior frontal ROI but there were no effects of group or the interaction between group and condition. For the contrast ‘Successful stop > Go’, there was a small effect of condition (more activation for the manual condition compared with the speech condition; β = -0.19; CI = -0.33 - -0.06).

In addition, small effects of condition were found for the contrast ‘Successful Stop > Go’ for the left (β = -0.17; CI = -0.28–0.07) and right (β = -0.16; CI = -0.27- -0.05) putamen, both showing more activity for the manual condition compared with the speech condition). No other effects were found for group or the interaction between group and condition.

Analysis of activity in the right IFG, SMA, and the control ROIs did not reveal differences between the conditions (manual, speech) or the groups (people who stutter, controls), nor an interaction between condition and group.

The inhibition literature demonstrates that the right IFC is selective for stopping responses (e.g., Xue et al., 2008). The whole-brain analyses show right lateralised activity in controls and bilateral activity in people who stutter for the contrast “Successful Stop > Go”. To test this statistically, we modelled side (left and right IFG (opercularis) ROIs) with effects of group, condition, and the interaction between them, with participant as a random factor. There was a small effect of condition, such that the manual task showed more activity compared with the speech task (β = -0.19, CI: -0.31,-0.07). There were no effects of group or side, suggesting that activity was not right lateralised for stopping behaviours. There were no interactions among these factors either.

Recently, the right IFG has been functionally segregated into five distinct parcellations, including reasoning, memory, attention, action, and perception. Analysing the rIFG as a single ROI may obscure important functional distinctions within the region. We, therefore, used the ROI parcellations from Hartwigsen et al. (2019) (provided by personal communication with the authors) to explore activity in this region further. We analysed the two parcellations relevant for addressing our hypotheses, namely the ROI for “action inhibition” (cluster 4 of Hartwigsen et al., 2019) and “action execution” (cluster 2 of Hartwigsen et al., 2019). Bayesian regression models were run in the same way as the previous ROI analyses (see Supplementary Materials and Supplementary Fig. S2). In sum, for Go trials in the “action inhibition” cluster, we found a small effect of group, with PWS having higher activity on average across both conditions relative to controls (β = 0.20, CI = 0.01 to 0.39). There was also weak evidence for a group by condition interaction (β = -0.28, CI = -0.56 to -0.01). Activity was lower in PWS for speech compared with the manual condition, but these two conditions evoked similar levels of activity in controls (see Fig. S2). All other analyses for data in this cluster and in the action execution cluster revealed no evidence for meaningful effects.

To summarise, these results indicate that the left inferior frontal gyrus was more activated when speaking compared to when making a button press, as expected, and that this effect was larger for controls compared with people who stutter. Otherwise, our region of interest analyses are consistent with our whole-brain findings that show no differences between conditions or groups. Considering the right IFG ROI as a whole, we find no differences between groups or task for any trial type nor the contrast ‘Successful Stop > Go’, which is inconsistent with the prediction made by the global inhibition hypothesis. When further parcellating the right IFG into relevant “action inhibition” and “action execution” clusters based on a previous analysis (Hartwigsen et al., 2019), we find weak support that the PWS group have greater activity on Go trials for the “action inhibition”, which could be considered supportive of the global inhibition hypothesis. However, there is a similar level of weak evidence in support of a group by condition interaction in this cluster, where the PWS group show lower levels of activity for the speech condition compared with the manual one, and controls activate this cluster to a similar extent in both conditions. This pattern is unexpected if the greater activity in PWS relative to controls is being driven by the manual rather than the speech condition. Given the level of support in these analyses is weak, and the large number of analyses being run, we suggest a cautious interpretation of these findings is warranted.

## 4 Discussion

We tested whether there were differences in the initiation and inhibition of motor responses in people who stutter and matched control participants in the context of the stop-signal paradigm. Participants completed the behavioural version of the stop signal reaction time (SSRT) task in the manual and speech domains (Xue et al., 2008), prior to repeating the tasks while fMRI was recorded from the whole brain. During the manual task, participants responded to a visual stimulus (left or right arrow) with their right index finger and during the speech task, participants read aloud a one-syllable pseudoword. On randomly inserted trials participants heard an auditory cue, which indicated they should inhibit, that is, stop their response. Previous work suggests that people who stutter have an overactive response inhibition mechanism (Neef et al., 2018). According to the global suppression hypothesis, shorter stop signal reaction times and hyperactivation of the right hemisphere inhibition network were expected to be associated with stuttering. Contrary to this prediction, in our behavioural study, people who stutter had longer reaction times on ‘go’ trials than controls but there was no significant difference in the speed of the stopping process estimated by the stop signal reaction time. This suggests that differences between the two groups lie in initiating a response rather than inhibiting an ongoing response and that this effect is more evident in the speech domain compared with the manual domain. The fMRI results revealed activation in the expected networks for each of the trial types; however, there were no differences in activity between the two groups.

Taken together, these results show subtle differences in initiating a response (behaviourally), but no differences either behaviourally or neurally between people who stutter and controls for response inhibition. We find no evidence in support of the idea that people who stutter have an overactive response inhibition mechanism. The behavioural and fMRI results are discussed below.

### 4.1 Behavioural results: differences in initiating but not inhibiting a response in people who stutter

Based on the findings that people who stutter have increased right IFC activity during speech and non-speech tasks (see reviews from Belyk et al., 2017; Chang & Guenther, 2020), and that right IFC activity has been linked with shorter reactive inhibition times (e.g., Aron & Poldrack, 2006), we predicted that people who stutter would have shorter stopping responses due to a constant heightened inhibition signal. If this is a domain general response, we should expect to see shorter stopping times for both manual and speech responses; an alternative hypothesis would be that this only occurs in the speech domain. Our results indicate that while people who stutter were slower to initiate a response (go reaction time) for both conditions, the stopping response (SSRT) was not different to that of controls. Our findings indicate that people who stutter do not differ in the time it takes to inhibit a response in either the manual or speech domains. The behavioural results of our study contrast with those from previous studies of people who stutter that also employed the manual stop signal task and found that people who stutter had longer stop signal reaction times than controls (Markett et al., 2016; Treleaven & Coalson, 2020). Our findings are in accord, however, with another study that found no differences between people who stutter and controls in a manual and verbal version of the SSRT task (Treleaven & Coalson, 2021). Further work is needed to reconcile these conflicting results, focusing on differences in methodology (see below). It is unlikely, however, that our failure to find a group effect for SSRT is due to a lack of power to detect differences. A previous study reported a moderate effect size of d = 0.61 (n = 28 per group) for the significant difference between groups for SSRT (Markett et al., 2016). In addition to no group differences in average measures of performance, examination of the distributions of SSRTs for our two groups shows they were very closely overlapping and our Bayesian analyses show strong evidence in favour of the null hypothesis for measures of the SSRT.

In contrast to the inhibition results, people who stutter had slower reaction times compared with controls on Go trials in the manual and speech domains. One explanation is that people who stutter have greater difficulty enacting a response under temporal uncertainty. For example, the previous study (Markett et al., 2016) used two tasks to estimate ‘go’ reaction times: one task had ‘go’ trials only and used fixed inter-trial intervals (500 ms), providing strong temporal predictability for when a ‘go’ response was required. The other task involved trials with varied inter-trial-intervals, which provide less temporal predictability. People who stutter only showed longer reaction times relative to controls when the timing of the trials was unpredictable (Markett et al., 2016). The authors suggest that this difference may be due to problems relying on internally generated timing compared with the externally generated timing provided by the predictability of the fixed inter-trial-intervals (Chang & Guenther, 2020; Markett et al., 2016). In the current study, a fixed inter-trial-interval of 2 s was used; however, ‘Go’ and ‘Stop’ trials were presented in a random order, which introduced temporal uncertainty. Therefore, this result is in accordance with previous work on temporal uncertainty difficulties in people who stutter and may be the result of an impairment in internal cueing, rather than a difference in higher-order cognitive control of responses. Further methodological differences make comparison between these studies difficult. The longer inter-trial-interval (2 s used here and 500 ms for Markett et al. (2016)) would allow the participant more time to prepare for the next trial, reducing task demands and allowing participants to respond more quickly, though note that in the current study, participants who stutter were slower to respond than controls. In addition, the Markett et al. (2016) study involved a semantic decision task (categorise and on-screen word as “animal” or “non-animal”), which has different cognitive demands compared with those of the speech task used in the current study. Even though the speech task used here might have been less taxing than the one used in the previous study, it is interesting to note that we still find the group of people who stutter had longer reaction times on ‘Go’ trials across both modalities than controls.

### 4.2 fMRI results: no group differences in brain activity during response initiation or inhibition

During ‘Go’ trials, both groups showed the expected patterns of brain activity for the manual and speech conditions, respectively. The manual condition evoked activity in a network of areas involved in execution of right index finger movement, including the left dorsal precentral gyrus, left putamen, SMA, and cerebellum bilaterally. In both groups, ‘Go’ trials in the speech condition, evoked activity bilaterally in the sensorimotor cortex, at the level of the face representation, SMA, insula, putamen, thalamus, and cerebellum. Consistent with the language demands of this task, there was left-lateralised activation of the inferior frontal cortex. Visually, the group of people who stutter had more spatially extensive task-evoked activation compared with the control group during both ‘Go’ and ‘Stop’ trials, which could reflect greater variability in regions recruited to this task in the stuttering group, which was larger than the control group. However, there were no group differences in the whole-brain analysis or when the analyses were constrained to pre-defined regions of interest in the motor inhibition network. There was weak evidence that people who stutter had greater task-evoked activity for ‘Go’ trials in a specific portion of the right IFG associated with action inhibition (Hartwigsen et al., 2019) but this effect was only present in the manual condition and not observed for speech. The ROI results confirmed that the left hemisphere IFG was more active for the speech condition compared with the manual condition, during ‘Go’, ‘Successful Stop’, and ‘Unsuccessful Stop’ trials, as expected. In sum, we find little evidence in support of differences between people who stutter and controls in terms of their brain activity when performing either a manual or a speech response to a cue, despite the behavioural finding of slower response initiation (‘Go’ reaction time).

The whole-brain fMRI results indicate that both groups activated the right IFG, frontal operculum and anterior insula more during ‘Successful stop’ responses compared with ‘Go’ responses, consistent with the idea that this region is selective for stopping behaviour in the brain (Aron & Poldrack, 2006; Aron et al., 2003, 2004, 2007; Xue et al., 2008). We found no difference, however, between activity in the left and right IFG ROIs . Therefore, we failed to replicate the previously reported finding of right-lateralised responses to stopping in right frontal regions.

While the right IFG has been a particular focus of the inhibition literature, it sits within a network of cortical-subcortical regions that carefully balance initiation and inhibition behaviour. During successful stop trials, participants also activated the putamen, postcentral gyrus, supramarginal gyrus, and cerebellum bilaterally, and the SMA extending to the cingulate motor area. These areas have been implicated in previous studies of motor inhibition. Both direct and indirect pathways from these cortical areas via the putamen project back to the cortex via the thalamus to balance excitatory and inhibitory control. The supramarginal gyrus was also activated in the inhibition of manual and spoken responses in previous work (Xue et al., 2008). Finally, the cingulate cortex is implicated in the cognitive control of inhibition. For example, the cingulate motor area was robustly activated (with a specific pattern for eye, hand or speech movement) when participants inhibited a congruent response in favour of an incongruent response (Paus et al., 1993). The cingulate cortex is also implicated in the suppression of tics (motor and verbal) Tourette’s syndrome (Jackson et al., 2021). Together, these prior findings highlight the role of the cingulate cortex in the control of the balance between the selection of motor responses and active suppression of others (Paus, 2001; Paus et al., 1993).

The lack of statistical difference between the groups during ‘stop’ trials suggests no difference in activation of brain areas involved in inhibitory control between people who stutter and controls. This result is inconsistent with the prediction that people who stutter would show overactivity in key regions of the stopping network. Alternatively, the DIVA model predicts that the right IFC is sensitive to error detection (Tourville & Guenther, 2010), and therefore might be more active in people who stutter during the “unsuccessful stop” stop trials. We find that both groups activated the rIFC and other hubs from the inhibition network; there was no difference between the groups, however. This is consistent with recent work showing no differences between children who stutter and those who do not using EEG to characterise error-related responses during a Go-No task (Liu et al., 2024). Such electrophysiological methods can give greater insight into temporally specific mechanisms compared to the fMRI technique used here. These results suggest that either people who stutter do not have an overactive error detection mechanism, or that the error studied in this paradigm (i.e. a response error) differs from the more subtle error detection involved in matching feedforward and feedback information during speech and stuttering. Furthermore, the current study attempted to measure global inhibitory responses, independent of speech and stuttering, by comparing manual and nonword responses (a *trait* effect). Our results find no evidence for a domain general difference in inhibitory responses in people who stutter, however we did not investigate connected speech or moments of stuttering (a *state* effect). Recent work using magnetoencephalography, which has greater temporal resolution than fMRI, reported that parts of the inhibition network (right pre-SMA) are active prior to speech onset and linked to slowed initiation time, particularly when speech is stuttered or stuttering is anticipated by the speaker (Liu et al., 2024). This result is in accord with our behavioural results which showed slower reaction times for people who stutter during the speech task. We did not find a neural correlate of this using fMRI, however. We also find a correlation between manual and speech ‘Go’ reaction times for the control group, but not for people who stutter, which is likely caused by longer reaction times for the speech task compared with the manual task (see Fig. 1, right panel).

Our failure to find the expected effects in the imaging part of the study could relate to the design of the study. There were a large number of ‘Go’ trials (144) but fewer ‘Stop’ trials (48) because the task requires stop trials to be unpredictable and in the minority. These ‘Stop’ trials are further divided into approximately 50% successful stops (~24) and 50% unsuccessful stops (~24). Another factor is variability within the groups. Even though our sample of 31 people who stutter was large for an imaging study, stuttering populations show considerable inter-individual differences (Yairi & Ambrose, 2013), including psycho-social differences, such as use of techniques to control stuttering, attitudes towards stuttering and anxiety, as well as differences in overt stuttering characteristics. Unexpectedly, fourteen participants (the majority of which were participants in the stuttering group) were excluded from the analyses based on not having at least 10/24 successful stop trials. This could be because the SSD value calculated from the behavioural experiment did not transfer well to the scanner for these participants (e.g., increased anxiety in the scanner, resulting in slower responses) and it is possible this was more of an affect for the stuttering participants.

Taken together across modalities, our behavioural results indicate the people who stutter show differences relative to controls in initiating a response, but not stopping a response but our imaging results indicate no differences in brain activity during these two different processes. Both the manual and speech tasks required cognitive control over movement in response to a cue. The functional imaging findings are not sensitive to differences in the neurochemical balance of inhibition and excitation. Magnetic resonance spectroscopy (MRS), on the other hand, can assess balances of metabolites that control the excitation (glutamate) and inhibition (GABA) of neuronal firing as well as neuronal health and integrity. These metabolites are particularly crucial to the fine balance of inhibition and excitation within basal ganglia motor circuits. To our knowledge, just one study has imaged the inhibition network in people who stutter using MRS (O’Neill et al., 2017). People who stutter were found to have lower NAA:Cr ratios (N-acetyl-aspartate plus N-acetyl-aspartyl-glutamate (NAA): Creatine) within the right inferior frontal cortex and white matter which is indicative of poorer neuronal health and density (Rae, 2014). In contrast, higher ratios were found in the posterior cingulate cortex, hippocampus, and thalamus. The NAA:Cr ratio also correlated with participants’ degree of stuttering (OASES, Section 1) for the left and right thalami. To assess excitation and inhibition more specifically, measures of glutamate and GABA ratios are required (Sears & Hewett, 2021). Further investigation into metabolite differences in people who stutter may help to understand differences in the initiation of speech between people who stutter and controls and further elucidate the underlying causes of stuttering.

## 5 Conclusions

Using a stop-signal reaction time task, we found that people who stutter were slower than controls to respond to simple ‘Go’ stimuli in both the manual and speech domains, but their stopping behaviour did not differ. Our fMRI results did not reveal any group differences in either task, nor for ‘Go’ and ‘Stop’ trial types. Overall, this study does not lend support to the hypothesis that the increased right IFC activity often reported in brain imaging studies of people who stutter reflects an overactive inhibitory response, and instead indicates subtle differences in the initiation of domain general responses in people who stutter.

## Ethics

The University of Oxford Central University Research Ethics Committee approved the study. Written informed consent was obtained from all participants for being included in the study, in accordance with the Declaration of Helsinki, and with the procedure approved by the committee.

## Data and Code Availability

The behavioural data that support the findings of this study are openly available on OSF; DOI 10.17605/OSF.IO/ZJSWA. The MRI data that support the findings of this study are openly available in Neurovault at https://identifiers.org/neurovault.collection:8669

## Author Contributions

**C.E.E.W.:** Conceptualisation, Methodology, Software, Resources, Formal Analysis, Investigation, Data Curation, Writing—Original Draft, Writing—Review & Editing, Visualisation, Supervision, Project administration, and Funding acquisition. **J. C.:** Investigation, Project administration, and Writing—Review & Editing. **S.K.:** Conceptualisation, Investigation, and Writing—Review & Editing. **G.J.C.:** Investigation, Writing—Review & Editing. **M.P.H.:** Investigation, Writing—Review & Editing. **K.E.W.:** Conceptualisation, Methodology, Writing—Review & Editing, Supervision, Project administration, and Funding acquisition.

## Declaration of Competing Interest

The authors have no financial, personal, or academic conflicts of interests.

## Supplementary Material

Supplementary Material
