# Supplementary material for: An fMRI study of initiation and inhibition of manual and spoken responses in people who stutter
Source: Imaging Neurosci (Camb). 2025 Jul 31;3:IMAG.a.89. doi: 10.1162/IMAG.a.89 (PMC12330837; doi:10.1162/IMAG.a.89)
Supplement: Supplementary Material [file IMAG.a.89_supp.pdf]

Supplementary Materials

Go Trials

Table S1. Activation peaks and coordinates for peaks in clusters significantly activated in the control group during 'go' trials for the manual condition. Cluster forming threshold Z > 3.1, extent threshold p < .05 corrected. The cluster size, peak Z statistic, and MNI coordinates of selected peaks are provided.

Cluster location	Number of voxels	z	X	Y	Z
Cortical and subcortical sensory and motor areas	12865				
L precentral gyrus		7.45	-34	-28	60
L postcentral gyrus		7.17	-40	-30	58
L putamen		6.63	-23	0	7
Supplementary motor area		6.52	-1	-2	53
L opercular cortex		5.58	-53	-21	17
R precentral gyrus		5.03	32	-9	57
L inferior frontal gyrus		4.79	-55	9	5
R inferior frontal gyrus		4.54	56	10	6
Cerebellum and occipital lobes	11471				
R cerebellum (VI)		7.89	24	-50	-26
R occipital pole		6.55	28	-95	-6
L cerebellum (VI)		6.93	-36	-44	-28
L occipital pole		6.57	-30	-94	-10
R cerebellum (VIII)		6.87	16	-60	-45
L cerebellum (VIII)		5.69	-30	-58	-55
Right cortical areas	1352				
R postcentral gyrus		5.88	56	-20	40
R planum temporale		4.75	54	-34	20
Right cortical and subcortical motor areas	1268				
R precentral gyrus		6.28	60	8	22
R putamen		5.51	28	6	2
R opercular cortex		4.72	46	8	2
Right lateral occipital cortex (superior)	158	4.2	28	-58	68

Table S2. Activation peaks and coordinates for peaks in clusters significantly activated in people who stutter during 'go' trials in the manual condition. Using Z>3.1 resulted in clusters of very large extent spanning multiple anatomical areas. We report locations for these clusters using Z>4.3 cluster-forming threshold for clarity. The cluster size, peak Z statistic, and MNI coordinates of selected peaks are provided.

Cluster location	Number of voxels	z	X	Y	Z
Cortical sensory and motor areas	11937				
L precentral gyrus		8.89	-38	-24	52
L postcentral gyrus		8.48	-41	-29	54
Supplementary motor area		8.11	-1	-3	54
L central opercular cortex		7.66	-53	-19	15
Paracingulate gyrus		7.62	4	14	46
Cingulate gyrus		7.16	-6	2	43
R frontal operculum cortex		6.91	36	16	10
R inferior frontal gyrus		6.89	58	11	5
L inferior frontal gyrus		6.56	-52	12	-3
R precentral gyrus		6.46	40	-2	60
Cerebellum and occipital lobe	11691				
R cerebellum (V/VI)		9.34	14	-52	-20
R occipital pole		8.28	33	-92	-4
R cerebellum (VIII)		7.93	27	-52	-55
L occipital pole		7.84	-25	-97	-10
L cerebellum (VI)		7.74	-31	-54	-25
L cerebellum (VIII)		6.93	-35	-50	-52
Left subcortical areas and opercular cortex	3845				
L putamen		8.59	-26	-4	4
L central opercular cortex		7.73	-44	-3	12
L thalamus		7.40	-13	-21	10
L caudate		6.97	-15	-4	18
Right subcortical areas and opercular cortex	2713				
R putamen		7.81	26	-2	4
R central opercular cortex		7.01	49	5	0
R caudate		5.82	12	11	6
R thalamus		5.32	11	-16	7
Right postcentral gyrus	1607	6.72	60	-16	38
Right lateral occipital cortex (superior)	359	5.98	12	-66	52
Right frontal pole	320	5.66	34	32	30
Right supramarginal gyrus	130	5.19	62	-36	24

Table S3. Activation peaks and coordinates for peaks in clusters significantly activated during 'go' trials in the control group during the speech condition. Cluster forming threshold Z > 4.3, extent threshold p < .05 corrected. The cluster size, peak Z statistic, and MNI coordinates of selected peaks are provided.

Cluster location	Number of voxels	z	X	Y	Z
Cerebellum and occipital lobe	5943				
L occipital pole		8.24	-26	-96	-6
R occipital pole		8.11	24	-96	-3
R cerebellum (VI)		7.31	20	-60	-25
L cerebellum (VI)		6.64	-17	-60	-21
R cerebellum (VIII)		6.51	14	-68	-51
L cerebellum (VIII)		6.17	-20	-66	-55
Cortical and subcortical motor areas	5898				
L postcentral gyrus		7.54	-44	-18	36
L Heschl's gryus		6.91	-51	-29	9
L planum temporale		5.41	-52	-36	20
L precentral gyrus		6.83	-52	-11	42
L inferior frontal gyrus (opercularis)		5.41	-55	12	-1
L putamen		6.00	-22	1	10
R putamen		6.30	23	0	9
L thalamus		6.24	-9	-16	10
R thalamus		5.14	12	-16	5
Right cortical areas	2690				
R postcentral gyrus		7.45	54	-8	38
R Heschl's gyrus		6.18	54	-9	5
R temporal pole		6.00	59	10	-5
R precentral gyrus		7.36	59	0	20
Supplementary motor area	500	6.56	-2	-2	72
Paracingulate gyrus	311	5.56	-7	16	40
Left cerebellum (VIII)	253	6.43	-16	-64	-54
Left precentral gyrus (superior)	67	5.76	-18	-32	60
Left frontal pole	60	5.67	-38	36	18
Left lateral occipital cortex (superior)	54	5.08	-26	-62	38
Right precentral gyrus (superior)	52	5.62	18	-30	62

Table S4. Activation peaks and coordinates for peaks in clusters significantly activated during 'go' trials in people who stutter during the speech condition. Cluster forming threshold Z > 3.1, extent threshold p < .05 corrected. The cluster size, peak Z statistic, and MNI coordinates of selected peaks are provided.

**Cluster location**	**Number of voxels**	**z**	**X**	**Y**	**Z**
Cortical and subcortical motor areas	20536				
L postcentral gyrus		8.49	-52	-10	28
R postcentral gyrus		8.36	54	-8	38
L precentral gyrus		7.50	-56	-8	46
R precentral gyrus		6.91	56	-5	45
L inferior frontal gyrus (opercularis)		6.17	-52	13	-1
L thalamus		6.59	-10	-16	5
R thalamus		5.85	12	-17	3
L putamen		5.86	-21	8	2
R putamen		5.52	25	12	-2
Cerebellum and occipital cortex	13344				
R occipital pole		8.39	24	-96	-4
L occipital pole		8.34	-24	-96	-12
R cerebellum (VI)		8.2	22	-62	-24
L cerebellum (VI)		7.28	-18	-61	-20
R cerebellum (VIII)		7.12	12	-70	-50
L cerebellum (VIII)		6.20	-16	-64	-55
Medial frontal cortex	3089				
Supplementary motor area		7.05	-4	0	62
L paracingulate cortex		5.75	-12	22	32
R cingulate cortex		5.91	5	14	40
Right precentral gyrus (superior)	254	5.68	20	-28	60
Left precentral gyrus (superior)	235	5.6	-18	-32	60
Temporal fusiform cortex	131	4.56	-38	-8	-36
Right lateral occipital cortex	125	4.99	30	-64	34
Left lateral occipital cortex	125	4.64	-28	-68	22
Left frontal pole	98	3.86	-42	42	30
Left lateral occipital cortex (superior)	97	4.04	-24	-64	54

Successful Stop Trials

Table S5. Activation peaks and coordinates for peaks in clusters significantly activated in the control group during 'successful stop' trials in the manual condition. Cluster forming threshold Z > 4.3, extent threshold p < .05 corrected. The cluster size, peak Z statistic, and MNI coordinates of selected peaks are provided.

Cluster Location	Number of	z	X	Y	Z
Medial and Left cortical and subcortical motor areas	10645				
L Planum temporale		7.63	-62	-32	14
L postcentral gyrus		7.17	-61	-18	25
L putamen		7.11	-21	4	6
L frontal opercular cortex		6.92	-36	19	6
L Heschl's gyrus		6.92	-52	-19	6
L central opercular cortex		6.89	-45	4	4
Paracingulate gyrus		6.68	-4	10	44
Supplementary motor area		6.42	-1	-1	55
L precentral gyrus (inferior)		6.36	-54	7	19
L lateral occipital cortex		6.06	-52	-62	6
L precentral gyrus (superior)		5.92	-35	-20	60
Cingulate gyrus		5.81	-2	18	33
L inferior frontal gyrus (opercularis)		5.72	-55	10	8
Right cortical and subcortical motor areas	6022				
R planum temporale		8.13	64	-32	18
R superior temporal gyrus		6.89	63	-26	2
R insular cortex		6.26	34	22	5
R inferior frontal gyrus (opercularis)		6.18	54	10	12
R Heschl's gyrus		6.06	51	-18	8
R lateral occipital cortex (anterior)		5.95	52	-62	3
R precentral gyrus		5.91	37	-2	61
R caudate		5.55	18	18	2
R putamen		5.38	26	5	4
R cerebellum	2555				
R cerebellum (VIII)		7.51	18	-68	-50
R cerebellum (VI)		7.37	33	-50	-28
Left cerebellum (VI)	783	7.10	-30	-56	-28
Left cerebellum (VII)	396	5.95	-30	-62	-50
Right lateral occipital cortex (posterior)	289	7.00	30	-92	-4

Right frontal pole	214	5.17	36	36	30
Left frontal pole	177	6.06	-28	44	26
Right intracalcarine cortex	150	5.42	14	-68	14
Left superior parietal lobe	98	5.44	-16	-56	60
Left thalamus	95	5.94	-8	-21	6
Right planum temporale	65	6.42	42	-6	-12
Left intracalcarine cortex	54	5.25	-16	-74	14
Right caudate	38	4.98	14	10	10
Right thalamus	3	4.65	8	-16	6

Table S6. Activation peaks for people who stutter during successful stop trials in the manual condition. Using Z>3.1 resulted in clusters of very large extent spanning multiple anatomical areas. We report locations for these clusters using Z>4.3 cluster-forming threshold for clarity. The cluster size, peak Z statistic, and MNI coordinates of selected peaks are provided.

Cluster Location	Number of voxels	z	X	Y	Z
Left and Right cortical and subcortical areas	31872				
L Planum temporale		9.18	-62	-32	14
R insular cortex		8.42	42	17	-2
Paracingulate gyrus		8.39	5	14	43
L insular cortex		8.25	-35	19	4
Supplementary motor area		8.05	-2	-4	56
L precentral gyrus		8.01	-30	-7	53
Cingulate gyrus		7.94	-6	6	43
L putamen		7.92	-24	2	6
L supramarginal gyrus		7.90	-56	-42	14
R putamen		7.84	-22	8	6
L parietal operculum cortex		7.77	-42	-36	17
R occipital pole		7.72	28	-96	-8
L occipital pole		7.65	-27	-96	-11
L postcentral gyrus		7.52	-38	-27	56
L inferior frontal gyrus (opercularis)		7.52	-52	10	-2
R caudate		7.21	15	12	4
R inferior frontal gyrus (opercularis)		6.81	50	14	12
R superior frontal gyrus		6.28	26	-4	53
R thalamus		5.67	10	-12	8
L caudate		5.04	-11	13	5
Cerebellum	5123				
R cerebellum (VI)		8.60	30	-49	-29
R cerebellum (VIII)		8.27	32	-51	-51
L cerebellum (VI)		7.74	-30	-54	-28
Right frontal pole (superior)	1067	6.68	34	44	24
Left cerebellum (VII)	679	7.07	-28	-64	-50
Left frontal pole	578	6.01	-38	38	30
Left thalamus	304	6.19	-10	-20	10
Right hippocampus	220	5.95	4	-24	-2
Right frontal pole (inferior)	72	5.70	26	46	-10
Left cingulate gyrus	57	6.22	-13	-30	40

Table S7. Activation peaks and coordinates for peaks in clusters significantly activated in the control group during 'successful stop' trials in the speech condition. Cluster forming threshold Z > 3.1, extent threshold p < .05 corrected. The cluster size, peak Z statistic, and MNI coordinates of selected peaks are provided.

Cluster Location	Number of	z	X	Y	Z
Left cortical and subcortical motor areas	9824				
L precentral gyrus		7.07	-42	-16	36
L Heschl's gyrus		7.07	-52	-20	6
L Planum temporale		6.96	-46	-30	8
L insula cortex		6.16	-31	26	2
L frontal operculum cortex		6.11	-36	14	8
L inferior frontal gyrus (opercularis)		5.94	-48	10	8
L thalamus		5.41	-12	-16	10
L putamen		5.28	-24	4	8
Right cortical and subcortical motor areas	9330				
R precentral gyrus		7.25	50	-6	28
R planum temporale		6.68	64	-32	16
R insular cortex		6.59	32	22	4
R putamen		5.13	24	2	8
R thalamus		5.11	19	-17	8
Occipital Pole and Cerebellum	7194				
L occipital pole		7.99	-26	-96	-6
R occipital pole		7.61	24	-98	-4
R cerebellum (VI)		6.23	18	-68	-20
L cerebellum (VI)		5.78	-20	-66	-19
R cerebellum (VIII)		5.19	12	-67	-44
Medial frontal cortex	3591				
Supplementary Motor Area		6.43	6	6	70
Paracingulate gyrus		6.09	-6	14	40
L cerebellum (VII)	362	5.18	-30	-62	-48
Right frontal pole	326	5.16	38	38	32
Left middle frontal gyrus	132	4.58	-26	-4	54
Left superior parietal lobe	106	4.02	-26	-56	44
Right intracalcarine cortex	104	4.70	16	-64	6
Left precentral gyrus (superior)	96	4.79	-18	-30	62

Table S8. Activation peaks and coordinates for peaks in clusters significantly activated in people who stutter during 'successful stop' trials in the speech condition. Cluster forming threshold Z > 3.1, extent threshold p < .05 corrected. The cluster size, peak Z statistic, and MNI coordinates of selected peaks are provided.

Cluster Location	Number of	z	X	Y	Z
Left cortical and subcortical motor areas	9581				
L frontal operculum		7.74	-36	16	6
L precentral gyrus		7.29	-58	0	28
L supramarginal gyrus (inferior)		7.26	-64	-42	22
L central opercular cortex		7.18	-48	6	2
L insula cortex		7.18	-32	26	2
L planum temporale		6.38	-41	-32	10
L inferior frontal gyrus (opercularis)		6.37	-50	13	-2
L putamen		5.67	-22	8	4
Right cortical and subcortical motor areas	8885				
R supramarginal gyrus		7.25	66	-40	14
R frontal operculum		7.24	32	18	12
R central opercular cortex		7.01	46	10	2
R postcentral gyrus		6.88	54	-8	38
R precentral gyrus		6.30	56	-4	48
R inferior frontal gyrus (opercularis)		5.91	52	14	2
R planum termporale		5.30	53	-24	10
Occipital Pole and Cerebellum	5777				
L occipital pole		8.20	-24	-94	-12
R occipital pole		7.44	26	-96	-4
L cerebellum (VI)		6.31	-32	-57	-28
R cerebellum (VI)		6.06	34	-56	-28
Medial frontal cortex	3876				
Supplementary Motor Area		7.52	6	-2	64
Paracingulate gyrus		6.90	6	10	48
Cingulate gyrus		6.04	-9	19	32
Right frontal pole	607	5.69	38	36	32
Right cerebellum (VIII)	552	5.75	10	-70	-52
Left cerebellum (VIII)	487	5.11	-30	-60	-58
Left frontal pole	295	4.67	-36	46	32
Left supramarginal gyrus (superior)	234	5.02	-44	-38	42
Left thalamus	223	5.05	-10	-16	6

Right thalamus	125	4.42	14	-12	8

Unsuccessful Stop Trials

Table S9. Activation peaks and coordinates for peaks in clusters significantly activated in the control group during 'unsuccessful stop' trials in the manual condition. Using Z>3.1 resulted in clusters of very large extent spanning multiple anatomical areas. We report locations for these clusters using Z>4.3 cluster-forming threshold for clarity. The cluster size, peak Z statistic, and MNI coordinates of selected peaks are provided.

Cluster Index	Number of voxels	z	X	Y	Z
Medial and left cortical motor areas	9884				
L frontal operculum cortex		7.57	-40	12	4
L postcentral gyrus		6.87	-40	-29	57
L precentral gyrus		6.18	-25	-9	60
Supplementary motor area		6.98	-1	-1	54
Paracingulate gyrus		6.86	-1	10	48
L central opercular cortex		6.74	-44	-5	14
Cingulate gyrus		6.37	9	25	30
L inferior frontal gyrus		5.93	-54	10	6
L planum temporale		5.83	-50	-33	10
Right cerebellum	2504				
R cerebellum (VI)		7.40	34	-50	-28
R cerebellum (VIII)		7.14	18	-66	-48
Right cortical areas	2502				
R supramarginal gyrus		7.14	64	-40	24
R planum temporale		5.71	60	-16	6
Right frontal regions	1195				
R insular cortex		7.53	42	18	-2
R inferior frontal gyrus (opercularis)		6.88	52	10	4
Left cerebellum (VI)	461	6.30	-34	-54	-28
Left thalamus	402	6.18	-10	-14	8
Left occipital pole	359	6.77	-32	-92	-12
Left cerebellum (VII)	320	5.97	-30	-60	-54
Right occipital pole	170	6.51	32	-92	-6
Right middle frontal gyrus	138	5.00	36	0	56
Right thalamus	70	5.80	10	-16	6
Left putamen	54	5.42	-22	4	8
Left lateral parietal lobe	50	4.99	-30	-58	60

Table S10. Activation peaks and coordinates for peaks in clusters significantly activated in people who stutter during 'unsuccessful stop' trials in the manual condition. Using Z>3.1 resulted in clusters of very large extent spanning multiple anatomical areas. We report locations for these clusters using Z>4.3 cluster-forming threshold for clarity. The cluster size, peak Z statistic, and MNI coordinates of selected peaks are provided.

Cluster Index	Number of [illegible]	z	X	Y	Z
Cortical and subcortical motor areas	16376				
R frontal operculum cortex		9.28	42	18	-2
R insular cortex		8.77	38	19	3
R inferior frontal gyrus (opercularis)		8.60	-52	10	4
Paracingulate gyrus		8.35	5	20	36
R supramarginal gyrus		7.78	64	-40	24
Supplementary motor area		7.45	-2	-3	54
Cingulate gyrus		7.58	-8	20	33
L precentral gyrus		7.94	-40	-20	53
R precentral gyrus		6.34	51	6	45
Left frontal cortex	2755				
L frontal operculum		9.26	-40	16	4
L insular cortex		8.72	-30	20	6
L inferior frontal gyrus		7.75	-52	12	-1
L putamen		5.10	-20	6	6
L caudate		4.66	-9	8	3
Right cortical areas	3824				
R supramarginal gyrus		7.78	64	-40	24
R planum temporale		6.71	57	-17	9
Right cerebellum	3352				
R cerebellum (VI)		8.17	26	-58	-22
R cerebellum (VIII)		7.90	18	-65	-50
Left cerebellum (VI)	814	7.51	-28	-56	-30
Left thalamus	776	6.90	-10	-20	9
Left cerebellum (VII)	243	6.71	-32	-62	-52
Right occipital pole	412	7.72	32	-92	-6
Right putamen	356	6.22	20	14	0
Right frontal pole	223	5.24	40	46	16
Lateral occipital cortex	188	5.39	46	-68	-12
Left frontal pole	135	5.23	-24	46	20
Left prefrontal gyrus (medial)	67	5.42	-12	-28	44

Table S11 Activation peaks and coordinates for peaks in clusters significantly activated in the control group during 'unsuccessful stop' trials in the speech condition. Cluster forming threshold Z > 3.1, extent threshold p < .05 corrected. The cluster size, peak Z statistic, and MNI coordinates of selected peaks are provided.

See Table 3 for details.

**Cluster Index**	**Number of voxels**	**z**	**X**	**Y**	**Z**
Right cortical and subcortical motor areas	11151	4.19	-32	48	30
R superior temporal gyrus		7.48	62	-16	0
R precentral gyrus		7.31	58	0	20
R Heschl's Gyrus		7.79	50	-20	8
R inferior frontal gyrus		5.92	52	24	-2
R central opercular cortex		6.80	50	8	-2
R supramarginal gyrus		6.45	52	-40	12
R insular cortex		7.11	34	24	4
R thalamus		5.35	11	-14	6
R putamen		5.71	25	1	10
Left cortical and subcortical motor areas	10508				
L Heschl's gyrus		7.79	-52	-20	8
L planum temporale		7.46	-42	-30	10
L precentral gyrus		7.32	-42	-16	36
L superior temporal gyrus		6.85	-65	-33	15
L inferior frontal gyrus		6.78	-51	11	0
L supramarginal gyrus		6.67	-64	-41	24
L insular cortex		6.50	-28	17	10
L thalmus		6.12	-10	-15	7
L putamen		5.22	-21	8	7
Occipital cortex and cerebellum	7458				
L occipital pole		7.61	-26	-96	-6
R occipital pole		7.33	22	-98	-4
L cerebellum (VI)		6.97	-24	-60	-24
R cerebellum (VI)		6.68	16	-60	-24
R cerebellum (VII)		5.64	11	-74	-44
L cerebellum (VII/VIII)		5.47	-32	-59	-52
Medial frontal cortex	3695				
Supplementary motor area		7.26	6	6	68
Paracingulate gyrus		6.79	0	16	44
Cingulate gyrus		6.55	8	18	37
Right frontal pole	302	4.86	26	58	28
Temporal pole	192	4.73	38	4	-42
Cingulate gyrus (posterior)	151	4.48	6	-24	26
Left frontal pole	81	4.82	-34	50	30

Table S12 Activation peaks and coordinates for peaks in clusters significantly activated in people who stutter during 'unsuccessful stop' trials in the speech condition. Cluster forming threshold Z > 3.1, extent threshold p < .05 corrected. The cluster size, peak Z statistic, and MNI coordinates of selected peaks are provided.

**Cluster Index**	**Number of voxels**	**z**	**X**	**Y**	**Z**
Right cortical speech motor areas	8324				
R supramarginal gyrus		7.73	66	-36	10
R superior temporal gyrus		7.15	58	-20	0
R inferior frontal gyrus		7.07	50	12	4
R precentral gyrus		7.01	54	-6	42
R temporal pole		6.82	51	13	-9
R frontal opercular cortex		6.90	38	24	2
R Heschl's gyrus		6.57	50	-20	7
Right cortical speech motor areas	8236				
L supramarginal gyrus		8.32	-66	-40	18
L planum temporale		7.91	-64	-32	12
L inferior frontal gyrus		7.49	-50	12	0
L Heschl's gyrus		7.39	-50	-22	8
L frontal opercular cortex		7.15	-36	16	8
L precentral gyrus		7.12	-42	-16	36
Left occipital cortex and cerebellum	3862				
L occipital pole		7.84	-24	-96	-12
L cerebellum (VI)		6.46	34	-54	-30
R cerebellum (VI)		6.43	28	-60	-26
Medial frontal cortex	3438				
Supplementary motor area		7.41	-4	6	54
Pre-supplementary motor area		7.12	10	6	66
Paracingulate		6.31	8	20	36
Cingulate		5.16	-9	19	32
Right occipital cortex	1005	7.06	24	-96	-4
Subcortical motor areas	778				
R thalamus		5.13	12	-12	4
R putamen		5.09	18	8	2
L thalamus		5.03	-10	-16	6
Right cerebellum (VII)	431	5.83	10	-74	-44
Left cerebellum (VII)	341	4.76	-20	-66	-54
Left frontal pole	338	4.19	42	50	28
Left putamen	186	5.19	-20	8	2
Left medial frontal gyrus	83	4.66	-30	-2	54

Successful stop > Go trials

Table S13. Activation peaks and coordinates for peaks in clusters significantly activated in the control group in the hand condition for the contrast Successful stop > Go. Cluster forming threshold Z > 3.1, extent threshold p < .05 corrected. The cluster size, peak Z statistic, and MNI coordinates of selected peaks are provided.

Cluster Index	Number of voxels	z	X	Y	Z
Right cortical areas	11329				
R planum termporale		8.36	58	-18	8
R supramarginal gyrus		8.12	64	-40	22
R superior temporal gyrus		7.82	58	-28	2
R Heschl's gyrus		7.76	52	-22	10
R insular cortex		6.77	42	16	-2
R inferior frontal gyrus (opercularis)		5.54	52	16	12
Left cortical areas	7996				
L planum temporale		7.90	-48	-28	8
L supramarginal gyrus		7.34	-62	-42	14
L insular cortex		7.18	-40	16	-4
L planum polare		5.96	-42	-8	-10
L inferior frontal gyrus (opercularis)		5.19	-47	12	15
Intracalcarine cortex	5244				
L intracalcarine cortex		6.43	-12	-74	12
R intracalcarine cortex		6.43	12	-66	10
Medial frontal cortex	2821				
Paracingulate cortex		6.21	0	28	34
R pre-supplementary motor area		5.69	16	10	60
Right frontal pole	873	4.64	32	44	30
Left frontal pole	766	4.80	-30	44	26
Precuneus cortex	500	4.84	2	-50	58
Left pre-supplementary motor area	458	4.62	-14	0	66
Cingulate gyrus	283	4.87	0	-16	28
Left cerebellum (VI)	184	4.56	-22	-70	-28

Table S14. Activation peaks and coordinates for peaks in clusters significantly activated in people who stutter in the hand condition for the contrast Successful stop > Go. Cluster forming threshold Z > 3.1, extent threshold p < .05 corrected. The cluster size, peak Z statistic, and MNI coordinates of selected peaks are provided.

Cluster Index	Number of voxels	z	X	Y	Z
Right cortical areas	11659				
R superior temporal gyrus		8.61	66	-26	8
R supramarginal gyrus		8.25	64	-42	20
R planum temporale		8.19	58	-18	8
R insular cortex		7.48	36	20	2
R Heschl's gyrus		7.19	50	-21	9
R temporal pole		6.53	52	9	-10
R inferior frontal gyrus (opercularis)		5.77	52	16	12
Left cortical areas	6941				
R planum temporale		8.21	-60	-34	12
R supramarginal gyrus		7.38	-64	-42	20
Intracalcarine cortex and precuneus	2885				
Precuneus cortex		5.37	-4	-46	50
R intracalcarine cortex		5.34	24	-62	4
L intracalcarine cortex		5.06	-12	-72	10
Medial frontal cortex	2192				
Paracingulate cortex		6.22	8	30	34
R pre-supplementary motor area		5.08	10	10	66
Left frontal pole	572	4.84	-28	52	18
Left superior frontal gyrus	332	5.00	-20	-2	66
Cingulate gyrus	134	4.22	4	-10	30

Table S15. Activation peaks and coordinates for peaks in clusters significantly activated in the controls group in the speech condition for the contrast Successful stop > Go. Cluster forming threshold Z > 3.1, extent threshold p < .05 corrected. The cluster size, peak Z statistic, and MNI coordinates of selected peaks are provided.

**Cluster Index**	**Number of voxels**	**z**	**X**	**Y**	**Z**
Right supramarginal gyrus	2047	6.01	60	-42	32
Medial frontal areas	931				
L pre-supplementary motor area		4.96	14	14	62
Paracingulate gyrus (anterior)		4.58	12	28	30
Paracingulate gyrus (posterior)		4.56	6	10	50
Right frontal areas	631				
R insular cortex		4.99	34	26	2
R opercular cortex		4.94	38	22	2
R inferior frontal gyrus (opercularis)		3.84	58	16	8
Left supramarginal gyrus	450	5.16	-58	-44	30
Right frontal pole	378	5.22	36	38	32
Left frontal pole	165	4.40	-34	36	32
Left superior frontal gyrus	155	4.59	-14	2	66

Table S16. Activation peaks and coordinates for peaks in clusters significantly activated in people who stutter in the speech condition for the contrast Successful stop > Go. Cluster forming threshold Z > 3.1, extent threshold p < .05 corrected. The cluster size, peak Z statistic, and MNI coordinates of selected peaks are provided.

Cluster Index	Number of voxels	z	X	Y	Z
Right temporal areas	2446				
R supramarginal gyrus		6.67	62	-42	32
R postcentral gyrus		4.31	62	-20	25
R planum temporale		4.21	62	-22	13
Right frontal areas	1420				
R insular cortex		6.73	44	22	-2
R inferior frontal gyrus (opercularis)		6.42	50	12	10
R frontal opercular cortex		6.37	34	18	10
Medial frontal areas	1412				
R superior frontal gyrus		5.89	18	6	66
Paracingulate gyrus (anterior)		5.37	8	30	34
Paracingulate gyrus (posterior)		4.84	6	10	47
Left supramarginal gyrus	1042	5.74	-58	-44	36
Left frontal areas	652				
L insular cortex		5.98	-34	18	-6
L frontal opercular cortex		5.86	-32	20	8
Right frontal pole	548	5.91	38	36	32
Right superior frontal gyrus	274	4.82	-14	-4	70
Right middle temporal gyrus	148	5.04	50	-26	-6
Right middle frontal gyrus	143	4.94	46	6	46
Right precuneus cortex	92	4.51	14	-70	38

Region of Interest Analysis

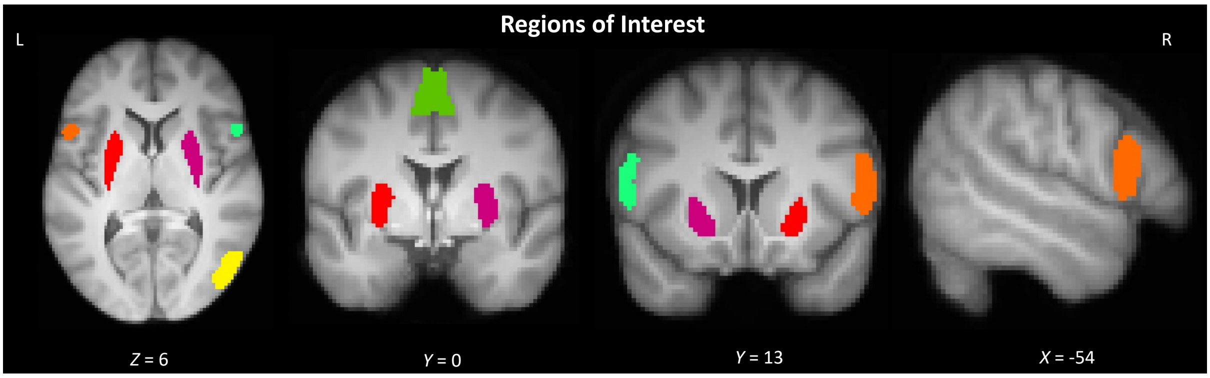

Figure S1. Binary masks created for the region of interest analysis. Masks were created using the Harvard-Oxford cortical and subcortical atlases (see "Imaging pre-processing and statistics" section for details). L = Left, R = Right. Green = SMA, Orange = Left IFG/operculum, Turquoise = Right IFG/operculum, Red = Left putamen, Purple = Right putamen, Yellow = Right lateral occipital cortex.

Function-Specific ROI analysis for the Right Inferior Frontal Gyrus

The right IFG has recently been parcellated into five separate clusters using large scale fMRI data (Hartwigsen et al., 2019). We used the ROI parcellations from Hartwigsen et al., (2019) (provided by personal communication with the authors) in order to explore our results further using these functional parcellations. We analysed the two parcellations that are relevant for addressing our hypotheses, namely the ROI for "action inhibition" (cluster 4 of Hartwigsen et al., 2019), and "action execution" (cluster 2 of Hartwigsen et al., 2019) (see figure S3).

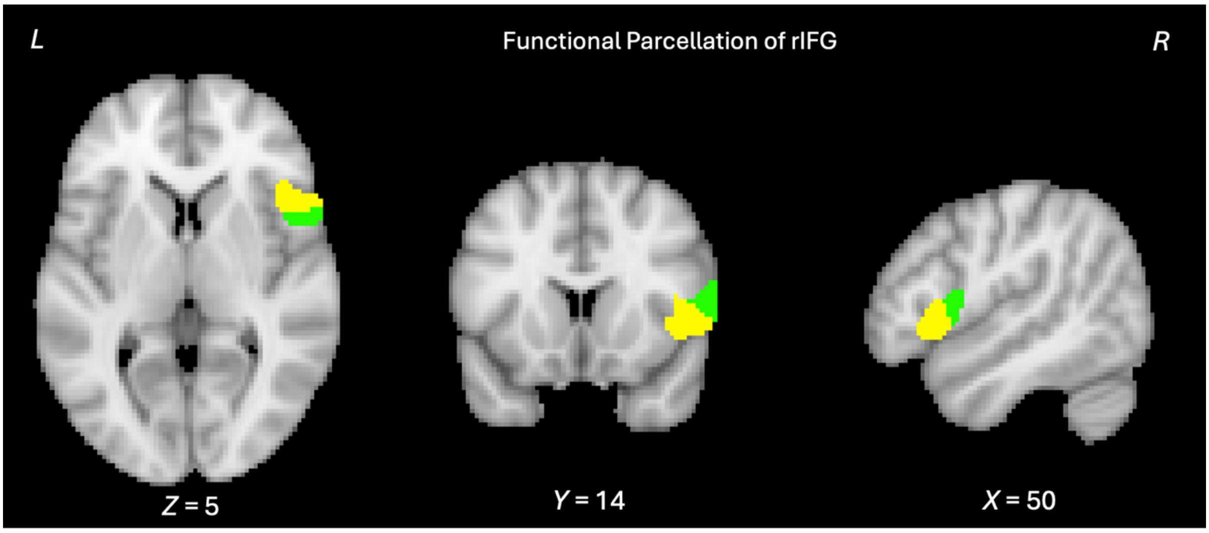

Figure S3. Binary masks created from the functional clusters of the right IFG (taken from Hartwigsen et al., 2019). L = Left, R = Right. Green = "Action Execution" (Cluster 2), Yellow = "Action Inhibition" (Cluster 4).

Featquery was used to extract mean percent signal change in these two clusters. The results were then subjected to the same regression models as used in the main ROI analysis, as follows:

Bayesian regression models were run separately for each ROI for the three trial types, GO, Successful Stop, and Unsuccessful Stop. Each model was run in the same way as our original ROI analysis, namely with factors of *group* (PWS, CON), *condition* (word, hand), and their *interaction*. *Participant* was included as a random factor.

The only evidence for effects came from the Action Inhibition cluster (cluster 4 of Hartwigsen et al., 2019) but was particularly weak. All other comparisons showed no evidence for meaningful effects.

Action Inhibition (cluster 4)

Analysis of Go trials revealed small effect for a *group*, with PWS showing higher activity on average than controls (β = 0.20, CI = 0.01 to 0.39). There was also a small effect for an interaction between *group* and *condition* (β = -0.28, CI = -0.56 to -0.01). There was no evidence for an effect of *condition* (β = 0.11, CI = -0.11 to 0.33).
Examination of the means (see Figure 1) suggest that the interaction between *group* and *condition* was due lower activation for word compared with hand in the group of people who stutter, while controls exhibited similar levels of activation for the two conditions, with slightly greater activation for word compared with hand. However, as the credible intervals only narrowly exclude zero, these effects should be interpreted with caution, as they indicate particularly weak evidence for a meaningful difference between contrasts.

For **Successful Stop trials**, no evidence of an effect was found for *group* (β = 0.08, CI = -0.11 to 0.27), *condition* (β = 0.03, CI = -0.10 to 0.16), or their *interaction* (β = -0.07, CI = -0.24 to 0.10).

For **Unsuccessful Stop trials**, no evidence of an effect was found for *group* (β = 0.08, CI = -0.11 to 0.27) *condition* (β = 0.03, CI = -0.10 to 0.16), or their *interaction* (β = -0.07, CI = -0.24 to 0.10)

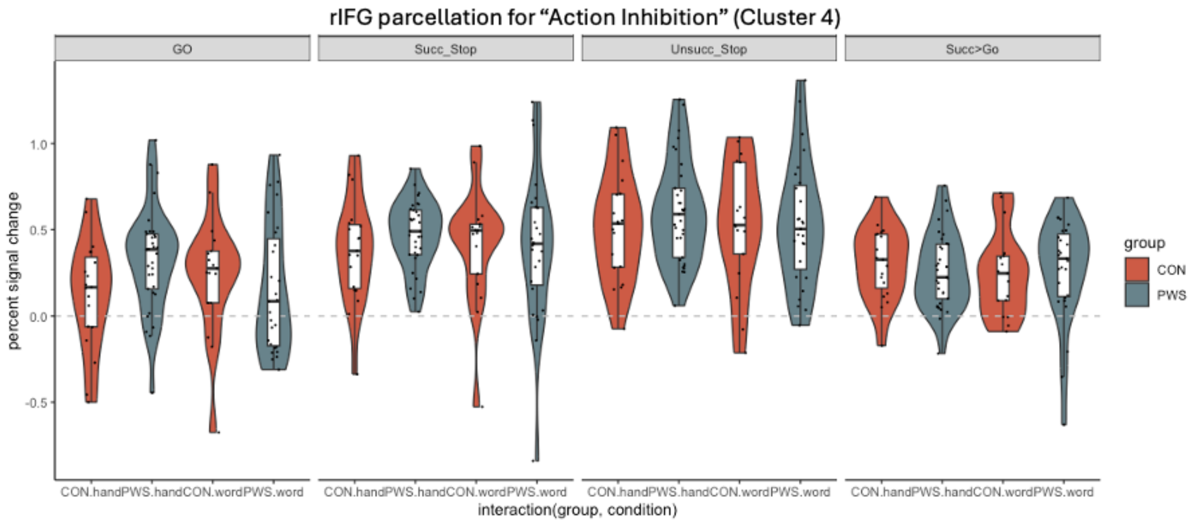

Figure S2. Percent signal change for the "Action Inhibition" (Cluster 4 from Hartwigsen et al., 2019). The group median is indicated by the solid black line quartiles by the white shaded box, whiskers show values within 1.5 times the IQR. Horizontal grey dotted line represents no signal change (0%).

Action Execution (Cluster 2)

Analysis of **Go trials** showed no evidence for a *group* effect (β = 0.06, CI = -0.13 to 0.24), a *condition* effect (β = 0.02, CI = -0.19 to 0.23), or an *interaction* between group and condition (β = -0.23, CI = -0.50 to 0.04).

For **Unsuccessful Stop trials, there was** no evidence of a *group* effect (β = 0.02, CI = -0.12 to 0.16), *condition* effect (β = 0.01, CI = -0.10 to 0.13), or an *interaction* between group and condition (β = -0.05, CI = -0.20 to 0.10).

For **Successful Stop trials**, there was no evidence of a *group* effect (β = -0.04, CI = -0.19 to 0.11), *condition* effect (β = -0.04, CI = -0.18 to 0.10), or an *interaction* between group and condition (β = 0.00, CI = -0.18 to 0.19).
